# Six new species of the braconid wasps of the genus *Aleiodes* Wesmael (Hymenoptera, Braconidae, Rogadinae) from the Korean Peninsula

**DOI:** 10.3897/zookeys.1283.193979

**Published:** 2026-06-24

**Authors:** Sergey A. Belokobylskij, Deokseo Ku, Hye-Rin Lee

**Affiliations:** 1 Zoological Institute of the Russian Academy of Sciences, St. Petersburg 199034, Russia Zoological Institute of the Russian Academy of Sciences St. Petersburg Russia; 2 The Science Museum of Natural Enemies, Geochang 50147, Republic of Korea The Science Museum of Natural Enemies Geochang Republic of Korea; 3 Gwacheon National Science Museum, Gwacheon, Republic of Korea Gwacheon National Science Museum Gwacheon Republic of Korea

**Keywords:** Asia, comparison, descriptions, Ichneumonoidea, parasitoids, subgenera, tribes

## Abstract

Six new species of the rogadine genus *Aleiodes* Wesmael, Aleiodes (Arcaleiodes) monochromus Belokobylskij & Ku, **sp. nov**., A. (Chelonorhogas) pseudalbitibia Belokobylskij & Ku, **sp. nov**., A. (Ch.) rufoniger Belokobylskij & Ku, **sp. nov**., A. (Aleiodes) crassicornis Belokobylskij & Ku, **sp. nov**., A. (A.) heterogamoides Belokobylskij & Ku, **sp. nov**., and A. (A.) imberbis Belokobylskij & Ku, **sp. nov**., are described and illustrated from the fauna of Korean Peninsula. A taxonomic key for the recognition of Palaearctic subgenera of the genus *Aleiodes* is provided.

## Introduction

The subfamily Rogadinae (Hymenoptera: Braconidae) is a cosmopolitan cyclostome taxonomic group of koinobiont parasitic wasps characterised by the mummification of its hosts, the micro- and macrolepidopteran caterpillars (Zaldívar-Riverón et al. 2008; Jasso-Martínez et al. 2021).

The tribe Aleiodini is one of eight known rogadine tribes, namely, Aleiodini, Betylobraconini, Clinocentrini, Facitorini, Gondwanocentrini, Rogadini, Stiropiini, and Yeliconini (Shimbori et al. 2024). This tribe includes only two genera, *Aleiodes* Wesmael, 1838 and *Heterogamus* Wesmael, 1838, but the number of species in this group is the largest among all rogadine groups (more than 630 species are already described) (Yu et al. 2016). The *Aleiodes* fauna of the tropical Asian and American continents is very rich and diverse (see, for example, Chen and He 1997; Shaw et al. 1997; Belokobylskij 2000; Fortier 2009; Butcher et al. 2012; Shimbori and Shaw 2014; Yu et al. 2016), but on the other hand, the information on the parasitoids of this genus in the North-East Palaearctic is incomplete and relatively scattered.

Several subgenera were distinguished in the genus *Aleiodes*, namely, *Aleiodes* s.str. (cosmopolitan), *Arcaleiodes* Chen & He, 1997 (East Palaearctic and Oriental regions), *Chelonorhogas* Enderlein, 1912 (cosmopolitan), *Eucystomastax* Brues, 1912 (Nearctic and Neotropical regions), *Hemigyroneuron* Baker, 1917 (Australasian, Afrotropical and Oriental regions), *Neorhogas* Szépligeti, 1906 (mostly Palaearctic region), and *Tetrasphaeropyx* Ashmead, 1889 (mostly Nearctic region) (van Achterberg 1991; Belokobylskij 2000; Yu et al. 2016). Unfortunately, the subgeneric division is practically not used in several recent publications, considering only the species groups (Fortier and Shaw 1999; Butcher et al. 2012; van Achterberg and Shaw 2016; van Achterberg et al. 2020). Nevertheless, we considered it is more convenient to use such a subgeneric division for the Palaearctic *Aleiodes* species, as it facilitates their determination because the division and the key to the possible *Aleiodes* species groups are not yet sufficiently developed.

Currently, information on the species of the genus *Aleiodes* of the Korean Peninsula has been published in several reviews, keys, and faunistic papers (for example, Papp 1985a, 1985b, 1989, 2003, 2018; Belokobylskij 2000; Ku et al. 2001; Lee et al. 2022a, 2022b, 2023; Yu et al. 2016), and approximately 45 species have been already recorded in this territory. In the present study, six species of *Aleiodes* from the subgenera *Arcaleiodes*, 1997, *Aleiodes* s.str., and *Chelonorhogas* are described from the South Korea as a new to science.

## Materials and methods

The terminology employed for the morphological features, sculpture, and body measurements follows Belokobylskij and Maetô (2009) and Belokobylskij et al. (2024). The wing venation nomenclature follows Belokobylskij and Maetô (2009) and Belokobylskij et al. (2024), with the terminology of van Achterberg (1993) shown in parentheses.

The specimens were examined using an Olympus SZ51 stereomicroscope. Photographs were taken with an Olympus OM-D E-M1 digital camera mounted on an Olympus SZX10 microscope (Zoological Institute of the Russian Academy of Sciences, St Petersburg, Russia). Image stacking was performed using Helicon Focus 8.0. The figures were produced using the Adobe Photoshop CS6 program.

The specimens (including types) examined in this study were deposited in the collections of the National Institute of Biological Resources (Incheon, Republic of Korea; **NIBR**), the Science Museum of Natural Enemies (Geochang, Republic of Korea; **SMNE**), and the Zoological Institute of the Russian Academy of Sciences (St Petersburg, Russia; **ZISP**). Abbreviation of the traps: **MT** – Malaise trap; **LT** – light trap. Abbreviations of the Korean provinces used in this paper as follows: **CB** – Chungcheongbuk-do; **CN** – Chungcheongnam-do; **GB** – Gyeongsangbuk-do, **GG** – Gyeonggi-do; **GN** – Gyeongsangnam-do, **GW** – Gangwon-do, **JB** – Jeollabuk-do, **JJ** – Jeju-do.

## Taxonomic account

### Class Hexapoda Blainville, 1816

**Order Hymenoptera Linnaeus, 1758**

**Family Braconidae Nees, 1811**

**Subfamily Rogadinae Foerster, 1863**

#### Genus Aleiodes

Taxon classificationAnimaliaHymenopteraBraconidae

Wesmael, 1838

58C06232-0339-58D7-A84F-0454D490BDD5

Aleiodes Wesmael, 1838: 194; Shenefelt 1975: 1163; Papp 1985a: 143; Shaw and Huddleston 1991: 95 (biology); van Achterberg 1991: 24; Belokobylskij 2000: 18; Zaldívar-Riverón et al. 2004: 225; Zaldívar-Riverón et al. 2008: 329 (phylogeny); Butcher et al. 2012: 6; van Achterberg and Shaw 2016: 8; Yu et al. 2016; van Achterberg et al. 2020: 12.
Petalodes Wesmael, 1838: 123; Shenefelt 1975: 1209; Tobias 1976: 90; van Achterberg 1991: 24 (as synonym of Aleiodes Wesmael, 1838); Belokobylskij 2000: 94 (as valid genus); van Achterberg and Shaw 2016: 8. [Type species: Petalodes unicolor Wesmael, 1838 (= Aleiodes compressor (Herrich-Schäffer, 1838)].
Nebartha Walker, 1860: 310; Shenefelt 1975: 1216; van Achterberg 1991: 24 (as synonym of Aleiodes). (Type species: Nebartha macropodides Walker, 1860).
Tetrasphaeropyx Ashmead, 1889: 634; Shenefelt 1975: 1260; Fortier and Sherman 2008: 445 (as subgenus of Aleiodes); Zaldívar-Riverón et al. 2008: 329. (Type species: Rogas pilosus Cresson, 1872).
Neorhogas Szépligeti, 1906: 605; Shenefelt 1975: 1205; van Achterberg 1991: 24 (as subgenus of Aleiodes); Belokobylskij 2000: 23; Zaldívar-Riverón et al. 2008: 329. [Type species: Neorhogas luteus Szépligeti, 1906 (= Rogas praetor Reinhard, 1863)].
Chelonorhogas Enderlein, 1912a: 258; Shenefelt 1975: 1187; van Achterberg 1991: 24 (as subgenus of Aleiodes); Belokobylskij 2000: 23; Zaldívar-Riverón et al. 2008: 329. [Type species: Chelonorhogas rufithorax Enderlein, 1912 (= A. convexus van Achterberg, 1991)].
Eucystomastax Brues, 1912: 223; Shaw 1993: 5 (as subgenus of Aleiodes); Zaldívar-Riverón et al. 2004: 225. [Type species: Eucystomastax bicolor Brues, 1912 (= Rogas melanopterus Erichson, 1848)].
Leluthinus Enderlein, 1912b: 96; Shenefelt 1975: 1202; van Achterberg 1991: 24 (as synonym of Aleiodes). (Type species: Leluthinus lividus Enderlein, 1912).
Aleirhogas Baker, 1917b: 383, 411; Shenefelt 1975: 1185; van Achterberg 1991: 24 (as synonym of Aleiodes). [Type species: Rhogas (Aleirhogas) schultzei Baker, 1917].
Hemigyroneuron Baker, 1917a: 284, 322; Zaldívar-Riverón et al. 2008: 329 (as subgenus of Aleiodes); Butcher and Quicke 2011: 1405; Butcher and Quicke 2015: 275. (Type species: Hemigyroneuron speciosus Baker, 1917).
Heterogamoides Fullaway, 1919: 43; Shenefelt 1975: 1188; van Achterberg 1991: 24 (as synonym of Aleiodes). (Type species: Heterogamoides muirii Fullaway, 1919).
Cordylorhogas Enderlein, 1920: 153; Shenefelt 1975: 1195; van Achterberg 1991: 31; Zaldívar-Riverón et al. 2004: 232; Zaldívar-Riverón et al. 2008: 329 (as synonym of subgenus Aleiodes). (Type species: Cordylorhogas trifasciatus Enderlein, 1920).
Hyperstemma Shestakov, 1940: 10; Shenefelt 1975: 1200; van Achterberg 1991: 24 (as synonym of Aleiodes). (Type species: Hyperstemma chlorotica Shestakov, 1940).
Dimorphomastax Shenefelt, 1979: 131; Shaw et al. 1998: 66 (as synonym of Aleiodes). [Type species: Dimorphomastax peculiaris Shenefelt, 1979 (= Aleiodes atriceps Cresson, 1869)].
Pholichora van Achterberg, 1991: 48; Quicke and Shaw 2005: 532; Zaldívar-Riverón et al. 2008: 329 (as synonym of Aleiodes); Butcher and Quicke 2011: 1405 (as synonym of subgenus Hemigyroneuron); Butcher et al. 2012: 9. (Type species: Hemigyroneuron madagascariensis Granger, 1949).
Arcaleiodes Chen & He, 1997: 60; Belokobylskij 2000: 23 (as subgenus of Aleiodes); Zaldívar-Riverón et al. 2008: 329; Butcher et al. 2012: 18. (Type species: Aleiodes unifasciatus Chen & He, 1991).
Vietorogas Long & van Achterberg, 2008: 313; Butcher et al. 2012: 15–17 (as synonym of Aleiodes). (Type species: Vietorogas bachma Long, 2008).

##### Type species.

(designated by Viereck 1914): *Aleiodes heterogaster* Wesmael, 1838 [= *Rogas albitibia* Herrich-Schäffer, 1838]. Type locality Belgium.

### Key to the Palaearctic subgenera of the genus *Aleiodes* Wesmael

**Table:** 

1	Ovipositor serrate ventrally, distinctly and relatively widely convex dorsally. Radial (marginal) cell of hind wing narrowed posteriorly to its basal third, then distinctly widened towards apex. Lateral carinae of scutellum complete and distinct. (Ovipositor sheath mostly glabrous, setae present only posteriorly and ventrally. Claws simple)	***Neorhogas* Szépligeti, 1906**
–	Ovipositor smooth ventrally, flat or only weakly convex dorsally. Radial (marginal) cell of hind wing subparallel or distinctly evenly widened towards apex. Lateral carinae of scutellum incomplete or absent	**2**
2	Radial (marginal) cell of hind wing not or rarely weakly widened towards apex, usually subparallel-sided. Mesopleuron, metapleuron, hind coxa and posterior tergites of metasoma often granulate. Inner (longest) spur of hind tibia ~ 0.3× as long as hind basitarsus	***Aleiodes* s.str**..
–	Radial (marginal) cell of hind wing distinctly widened towards apex, sometimes at least in distal half. Mesopleuron, metapleuron, hind coxa and often posterior tergites of metasoma without granulation, often smooth or reticulate-rugose. Inner (longest) spur of hind tibia usually ~ 0.40× (rarely 0.35×) as long as hind basitarsus	**3**
3	Second abscissa of mediocubital vein (1-M) of hind wing distinctly convex. Medial (basal) cell of hind wing rather narrow, 6.0–7.0× longer than its width. Basal (1-M) and recurrent (m-cu) veins of fore wing distinctly divergent posteriorly. (Claw simple. Antenna of female often with ring of white segments)	***Arcaleiodes* Chen & He, 1997**
–	Second abscissa of mediocubital vein (1-M) of hind wing straight. Medial (basal) cell of hind wing rather wide, 4.3–5.5× longer than its width. Basal (1-M) and recurrent (m-cu) veins of fore wing usually not divergent posteriorly	***Chelonorhogas* Enderlein, 1912**

#### Aleiodes (Arcaleiodes) monochromus

Taxon classificationAnimaliaHymenopteraBraconidae

Belokobylskij & Ku sp. nov.

3E577B02-7EC5-529A-82E9-857E033E66D5

https://zoobank.org/5712D2E7-3DE9-4BA4-878D-891987CF5C49

Figs 1, 2

##### Type material.

***Holotype*** • female, South Korea [GN] Goseong-gun, Sangri-myeon, Museon-ri, Mt. Muisan (Munsuam), LT, 5–6.VI.1995 (Jesik Jeon leg.) (NIBR). ***Paratypes***: South Korea **[GG]** • Seoul-si, Dongdaemun-gu, 57 Hoegi-ro, Hongreung Arboretum National Institute of Forest Science, MT, coniferus forest, 1–30.VI.2024 (Deokseo Ku, Yong Hwan Park), 1 female (ZISP) • Yangpyeong-gun, Okcheon-myeon, Yongcheon-ri, Mt. Yongmunsan, Yangpyeong Youth Training Center, LT, 28–29.VII.2000 (Tae-Ho An), 1 male (SMNE). **[CB]** • Jecheon-si, Susan-myeon, Jeongok-ri, San 30, LT, 15.VI.2024 (Hyung-Keun Lee), 1 male (SMNE) • Danyang-gun, Jeokseong-myeon Sang-ri, San 17-4, LT, 15.VI.2024 (Hyung-Keun Lee), 1 male (ZISP). **[GB]** • Uiseong-gun, Geumseong-myeon, Sujeong-ri, Mt. Bibongsan, sweeping, 7.V.1999 (collector unknown), 1 female (SMNE). **[GN]** • Ulsan-si, Ulju-gun, Sangbuk-myeon, Icheon-ri, Mt. Sinbulsan, LT, 31.VII–1.VIII.2003 (Tae-Ho An), 3 males (SMNE, ZISP) • Hamyang-gun, Macheon-myeon, Samjeong-ri, Mt. Jirisan, Chotdaebong-peak, 12–13.VII.2002 (Tae-Ho An), 1 male (SMNE) • Goseong-gun, Gaecheon-myeon, Mt. Yeonhwasan, Temple Okcheonsa, LT, 27–28.V.1995 (Jungseok Park), 1 male (SMNE) • Goseong-gun, Sangri-myeon, Museon-ri, Mt. Muisan (Munsuam), LT, 2–3.VI.2000. (Byeonghyeon Mo), 1 male (SMNE) • same label, but (Tae-ho An), 1 female (SMNE) • Goseong-gun, Sangri-myeon, Osan-ri, Mt. Odusan, LT, 6–7.VIII.1999 (Jesik Jeon), 1 female (SMNE) • Geoje-si, Dongbu-myeon, Buchun-ri, (Observatory), LT, 24–25.VI.1994 (Jungseok Park), 1 male (SMNE) • same locality, LT, 25–26.VI.1994 (Jesik Jeon), 1 male (ZISP); • Goseong-gun, Hail-myeon, Suyang-ri, 34°58'34.8"N, 128°12'08.3"E, 18.VI.2022 (S. Belokobylskij), 1 male (ZISP) • Yang-ri, Dalseong-gun, Daegu, 35.71331°N, 128.5114°E, sweeping, 5, 11.VIII.2024 (S. Belokobylskij), 1 female (ZISP) • same locality, 9–11.VII.2025 (E. Tselikh), 1 male (ZISP); Jahye-ri, Geumseo-myeon, Sancheong-gun, 35.429560°N, 127.8126°E, sweeping, 29.VI 2025 (S. Belokobylskij), 1 female (ZISP). **[JJ]** • Eoseungsaengak, Haeando, Jeju-si, Jeju-do, MT, 9–22.VII.2017 (collector unknown), 1 female (SMNE).

**Figure 1.**
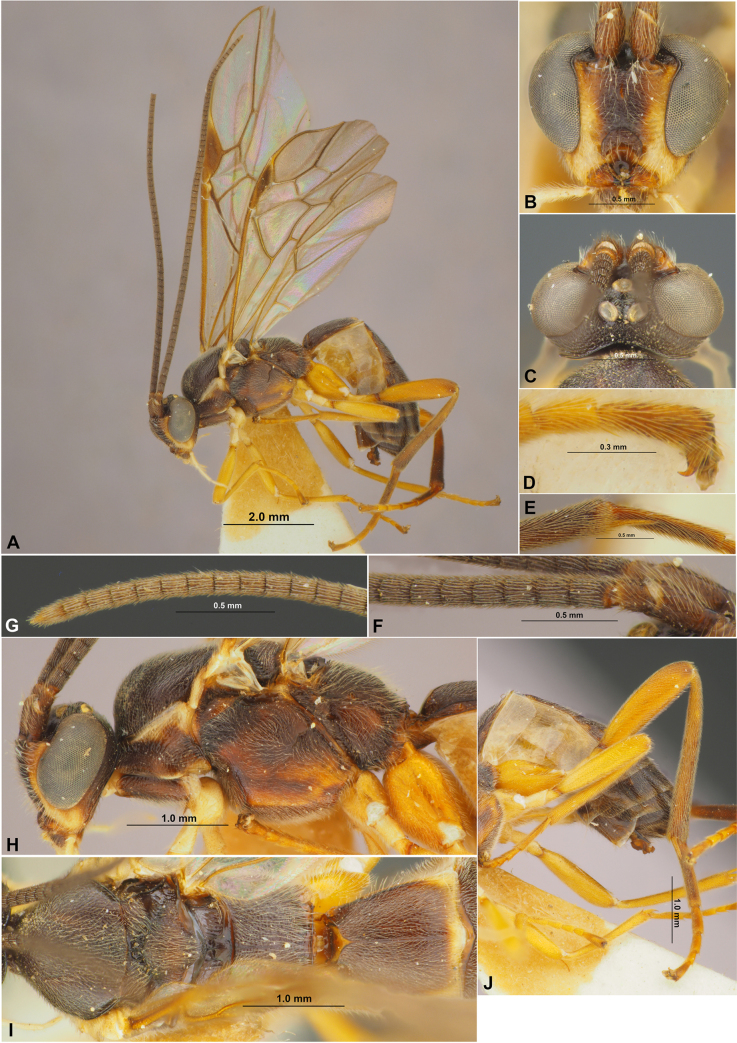
Aleiodes (Arcaleiodes) monochromus sp. nov., female, holotype. **A**. Habitus, lateral view; **B**. Head, front view; **C**. Head, dorsal view; **D**. Claw of hind leg; **E**. Tibial spurs and basitarsus of hind leg, inner side; **F**. Basal segments of antenna; **G**. Apical segments of antenna; **H**. Head and mesosoma, lateral view; **I**. Mesosoma and first metasomal tergite, dorsal view; **J**. Hind leg.

**Figure 2.**
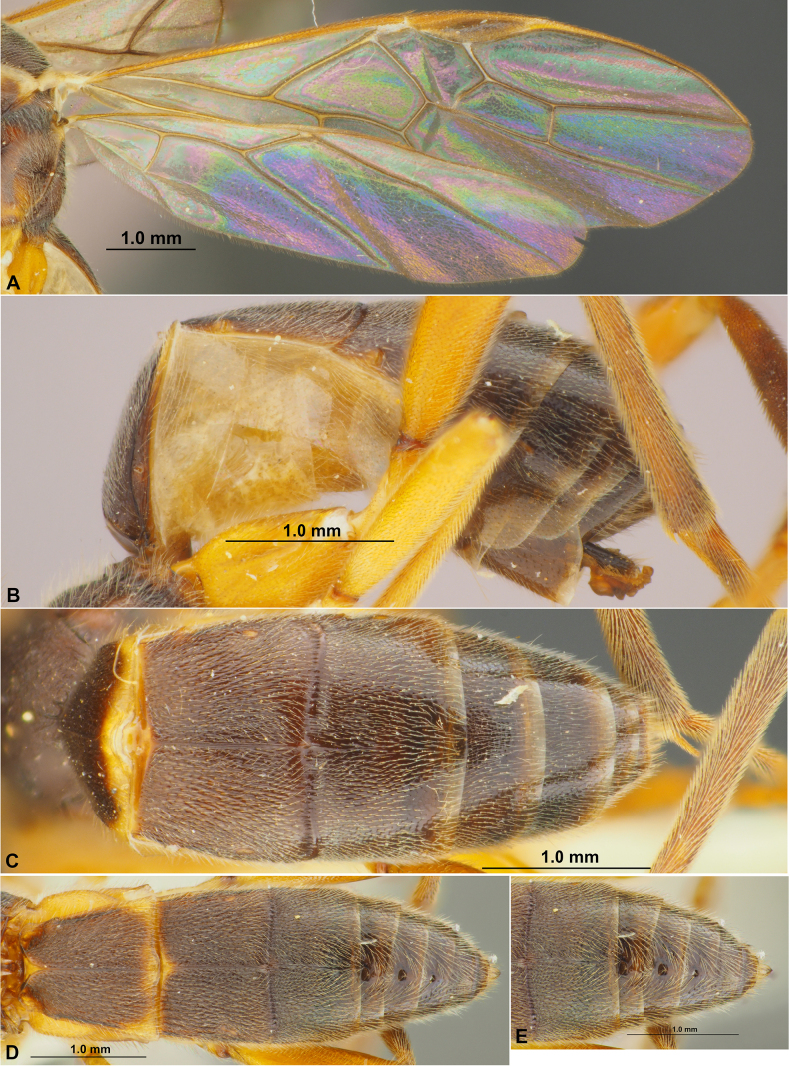
Aleiodes (Arcaleiodes) monochromus sp. nov., female, holotype (**A**–**C**) and male, paratype (**D**, **E**). **A**. Wings; **B**. Metasoma, lateral view; **C, D**. Metasoma, dorsal view; **E**. Posterior part of metasoma.

##### Description.

**Female. *Body*** length 8.5–9.5 mm; fore wing length 6.6–7.7 mm.

***Head*** width (dorsal view) 1.8–2.0× its median length, 1.1× width of mesoscutum. Occipital carina complete and rather distinct or sometimes fine dorsally, curved towards ocelli. Head behind eye (dorsal view) strongly and almost linearly narrowed; transverse diameter of eye 3.0–3.7× length of temple. Ocelli enlarged, all three ocelli same size, arranged in almost equilateral triangle; POL 0.4–0.5× Od, 1.0–1.5× OOL; Od 2.5–3.0× OOL. Eye high, bean-shaped, 1.6–1.7× higher than broad (lateral view). Face weakly convex, its width 0.9–1.1× height of face and clypeus combined, 0.6–0.7× height of eye. Hypoclypeal depression relatively small, circular, its width 1.0–1.2× distance from edge of depression to eye, ~ 0.4× minimum width of face. Malar space ~ 0.2× height of eye, 0.7–0.8× basal width of mandible. Head below eyes (front view) distinctly and weakly-curvedly narrowed. ***Antennae*** slender, setiform, 61–64-segmented. Scape relatively long, 1.7–2.0× longer than its maximum width. First flagellar segment 1.5–1.8× longer than its apical width, 1.2–1.3× longer than second segment. Medial segments weakly elongate. Penultimate segment 1.7–1.8× longer than wide. Apical segment with distinct and rather long ‘spine’.

***Mesosoma*** 1.7–1.8× longer than high. Mesoscutum approximately as wide as its medial length (dorsal view), highly and curvedly elevated above pronotum (lateral view). Prescutellar depression (scutal sulcus) relatively short, distinctly curved, 0.20–0.25× as long as scutellum, with four–five distinct carinae, rugulose between carinae. Scutellum without lateral carinae. Precoxal sulcus indistinct. Metanotum with very low and obtuse dorsal tubercle (lateral view). Propodeum evenly convexly curved posteriorly, without lateral tubercles.

***Wings***. Fore wing 3.0–3.3× longer than its maximum width. Radial vein (r) arising before middle of pterostigma. Radial (marginal) cell not shortened; metacarp (1-R1) 1.6–1.7× longer than pterostigma. First radial abscissa (r) 1.1–1.5× maximum width of pterostigma. Second radial abscissa (3-SR) 1.7–1.9× longer than first abscissa (r) and formed obtuse angle with it, 0.30–0.35× as long as third radial abscissa (SR1), 1.5–1.8× longer than first radiomedial vein (2-SR). First radiomedial vein (2-SR) distinctly curved in posterior half; second radiomedial vein (m-cu) weakly inclivous. Second radiomedial (submarginal) cell medium length, not narrowed distally, 2.6–3.1× longer than its maximum width, approx. as long as brachial (subdiscal) cell. Recurrent vein (m-cu) straight, 1.6–2.1× longer than second abscissa of medial vein (2-SR+M), approx. as long as first radiomedial vein (2-SR). Nervulus (cu-a) curved, subperpendicular, distinctly postfurcal, distance (1-CU1) between basal vein (1-M) and nervulus (cu-a) almost 2.0× longer than nervulus (cu-a); first abscissa of cubital vein (1-CU1) 0.7× the curved second abscissa (2-CU1). Brachial (subdiscal) cell wide, distinctly curved on anterior and distal margins. In hind wing, radial (marginal) cell distinctly widened distally. Radial vein (SR) arising from costal vein (2-SC+R) not far from basal vein (r-m). Nervulus (cu-a) weakly curved. First abscissa of mediocubital vein (M+CU) 1.2–1.3× longer than curved second abscissa (1M). Recurrent vein (m-cu) completely absent.

***Legs***. Hind trochantellus 1.8–2.0× longer than hind trochanter (measured on their lower margins). Hind femur 3.8–4.0× longer than wide. Longest inner spur of hind tibia weakly sinuate, 0.4–0.5× as long as hind basitarsus. Inner distal margin of hind tibia without comb of dense pale setae. Hind tarsus approx. as long as hind tibia. Hind basitarsus 0.7–0.8× as long as second to fifth segments combined. Second segment of hind tarsus 0.40–0.45× as long as basitarsus, 1.1–1.3× longer than fifth segment (without pretarsus). Claw simple, not pectinate, and without bristles.

***Metasoma***. Segments behind fourth tergite distinctly protruding. First tergite distinctly and almost evenly linearly widened towards apex, not strongly widened basally; length of tergite 1.10–1.15× its posterior width; posterior width 2.0–2.3× its anterior (basal) width. Basal area of first tergite rather wide and elongate, with singly pointed medial break posteriorly. Medial length of second tergite 0.8–0.9× its basal width, 1.10–1.15× length of third tergite; its smooth basal area short, but distinct. Suture between second and third tergites shallow, weakly sinuate and crenulate. Ovipositor sheath 0.3× as long as hind basitarsus, ~ 0.25× as long as first tergite.

***Sculpture and pubescence***. Vertex densely granulate with fine reticulation; frons partly almost smooth and finely reticulate-granulate; temple entirely densely granulate-reticulate; face densely transverse and partly curvedly striate with very dense fine additional rugulosity between striae. Mesoscutum entirely densely granulate with fine reticulation partly, with fine undulate rugosity (sometimes very fine) in medio-posterior third. Scutellum densely granulate. Mesopleuron rather finely and densely punctate, space between punctulae smooth or partly finely coriaceous; posteriorly with almost smooth small area. Propodeum entirely distinctly densely granulate-reticulate, with distinct and complete medial carina. First and second metasomal tergites entirely densely and relatively finely striate and with dense rugulosity between striae, their basal areas smooth, with distinct and complete medial carinae; third tergite densely reticulate-coriaceous with punctation, with fine medial carina only basally. Following tergites densely and finely punctate-coriaceous.

***Colour***. Head mostly black, face laterally (sometimes only upper) and below and malar space pale reddish brown, yellow or even cream-yellow. Mesosoma mostly black, sometimes laterally mostly dark reddish brown, lateral part of pronotum with narrow yellow stripes dorsally and ventrally (at least posteriorly). Metasoma mostly black, first tergite narrowly posteriorly and metasoma in lower basal half yellow. Antennae entirely black or dark brown. Palpi pale yellow or yellow. Tegula pale yellow or creamy yellow. Fore and middle legs pale brown to yellowish brown; hind legs pale brown or yellowish brown in basal half, hind tibia and tarsus brown to dark brown, partly paler. Wing subhyaline; pterostigma entirely dark brown.

**Male**. Body length 7.0–8.5 mm; fore wing length 5.1–5.6 mm. Antennae 52–59-segmented. Medial length of first tergite 1.15–1.20× its posterior width. Third tergite anteriorly narrowly pale yellow. Third tergite sometimes with medial carina at least in basal half or complete. Side of pronotum mostly creamy yellow. Metasomal tergite behind third one with distinct oval submedial holes of tergal glands. Otherwise similar to female.

##### Comparative diagnosis.

This new species takes an intermediate position according to the known keys of the Palaearctic and Oriental species from this subgenus (He et al. 2000; Belokobylskij 2000; Butcher et al. 2012) and differs from all known species by monochrome dark brown or black colouration of the antenna as well as predominantly dark brown to black colour of the body (including metasoma). *Aleiodes monochromus* sp. nov. is similar to A. (Arc.) hubeiensis (Chen & He, 1997) from China (Hubei Province) (Chen and He 1997), but differs from the latter (besides colour of antenna) by having the first and second metasomal tergites mostly black, tibia of the hind legs mostly dark brown or black, and maxillary palp creamy yellow. The new species also differs from A. (Arc.) arsenjevi (Belokobylskij, 1988) from the Russian Far East and Korea (Belokobylskij 1988) by smaller diameter of the hind ocellus (Od 2.5–3.0× OOL), the second tergite lacking yellow stripes, the first tergite 1.1–1.2× longer than its posterior width, and the evenly dark colouration of the antenna.

##### Etymology.

Named from *monochromus* (Greek for monochrome), referring to the evenly dark colouration of the antenna.

##### Host.

Unknown.

##### Distribution.

Korean Peninsula.

#### Aleiodes (Chelonorhogas) pseudalbitibia

Taxon classificationAnimaliaHymenopteraBraconidae

Belokobylskij & Ku sp. nov.

627BAFBA-665B-5CB9-8E83-4B797CFFE5CE

https://zoobank.org/AE6449D8-2690-4059-9D40-98212AAABC18

Figs 3, 4

##### Type material.

***Holotype*** • female, South Korea, “Korea (GB), Andong-si, Bukhu-myeon, Daehyeon-ri, 22.VI–29.VI.2022, Gi-Myon Kwon (Malaise trap)” (NIBR). ***Paratypes*: [CB]** • 1240-1 (Daeso), Jungdong-myeon, Soi-myeon, Eumseong-gun, LT, 29.VI.2024 (Hyung-Keun Lee), 1 female (SMNE). **[CN]** • Seocheon-gun, Maseo-myeon, National Institute of Ecology, MT, 36°01'47.19"N, 126°43'35.77"E, 16.VI–5.VII.2017 (Hyung-Keun Lee), 2 females (SMNE, ZISP). **[GB]** • same label as in holotype, 1 female (SMNE) • same label as in holotype, but 18.V–2.VI.2022, 1 male (ZISP) • Gimcheon-si, Guseong-myeon, Heungpyeong-ri, MT, 36°04'58.6"N, 128°30'30.48"E, 4–18.VII.2017 (Hyung-Keun Lee), 1 female (SMNE) • Juwangsan Mountain, Cheongsong-gun, 25.V.1989 (collector unknown), 1 male (SMNE). **[GG]** • Suwon, Mt. Yeogi, MT (Matsumura), 10.VII.1995 (June-Yeol Choi), 1 female (SMNE) • same label, but 3.VII.1995, 2 females (SMNE) • Suwon, Mt. Yeogi, MT (Ye/B1), 30.VI.1997 (June-Yeol Choi), 1 female (ZISP) • Sucheong-dong, Osan-si, Gyeonggi-do, Forestry Research Institute, LT, 22.VI.1999 (Hyung-Keun Lee), 1 female (SMNE). **[GN]** • Jinju-si, Ilbanseong-myeon, Gaseon-ri, MT, 21.V–6.VI. 2022 (Tae-Ho An), 1 female (ZISP) • Goseong-gun, Jangpal-ri, 35°40'12"N, 127°53'17.8"E, 1.VII.2023 (S. Belokobylskij), 1 female (ZISP). **[JN]** • Hwasun-gun, Chunyang-myeon, Gabong-ri, 34°47'64"N, 126°57'49.37"E, MT, 19.VI–3.VII.2017 (Hyung-Keun Lee), 1 female (SMNE); same label, but 9–19.VI.2017, 1 female (ZISP).

**Figure 3.**
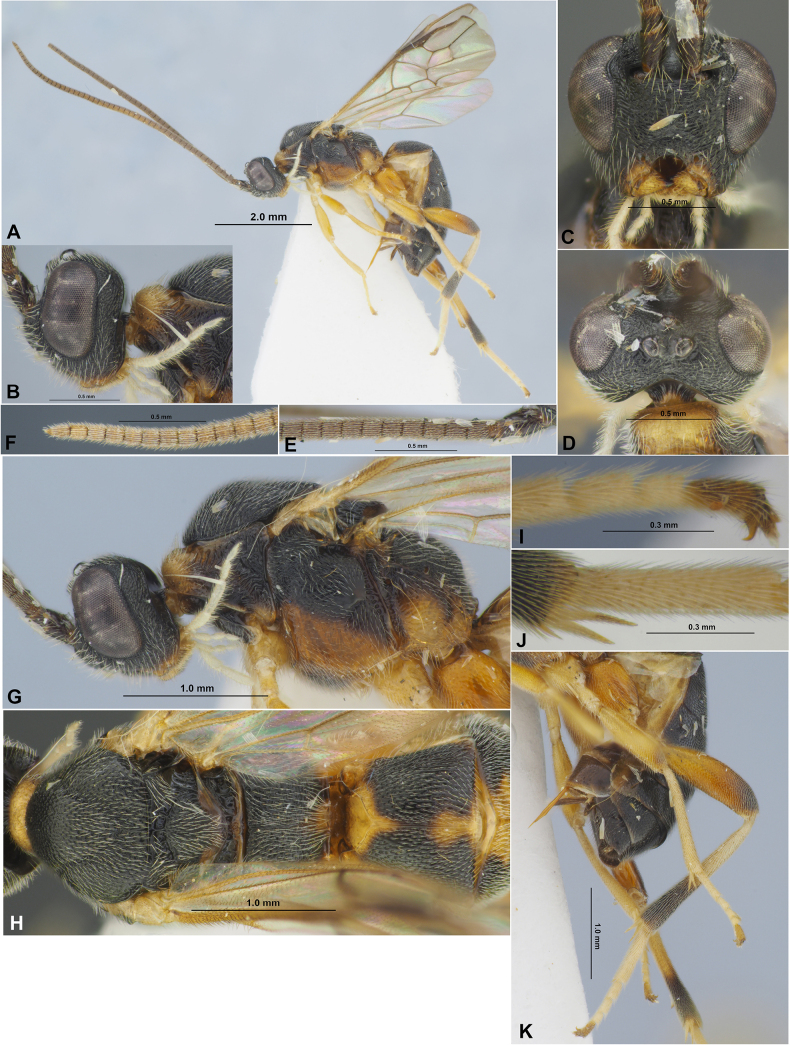
Aleiodes (Chelonorhogas) pseudalbitibia sp. nov., female, holotype. **A**. Habitus, lateral view; **B**. Head, lateral view; **C**. Head, front view; **D**. Head, dorsal view; **E**. Basal segments of antenna; **F**. Apical segments of antenna; **G**. Head and mesosoma, lateral view; **H**. Mesosoma and first metasomal tergite, dorsal view; **I**. Claw of hind leg; **J**. Tibial spurs and basitarsus of hind leg, inner side; **K**. Hind leg.

**Figure 4.**
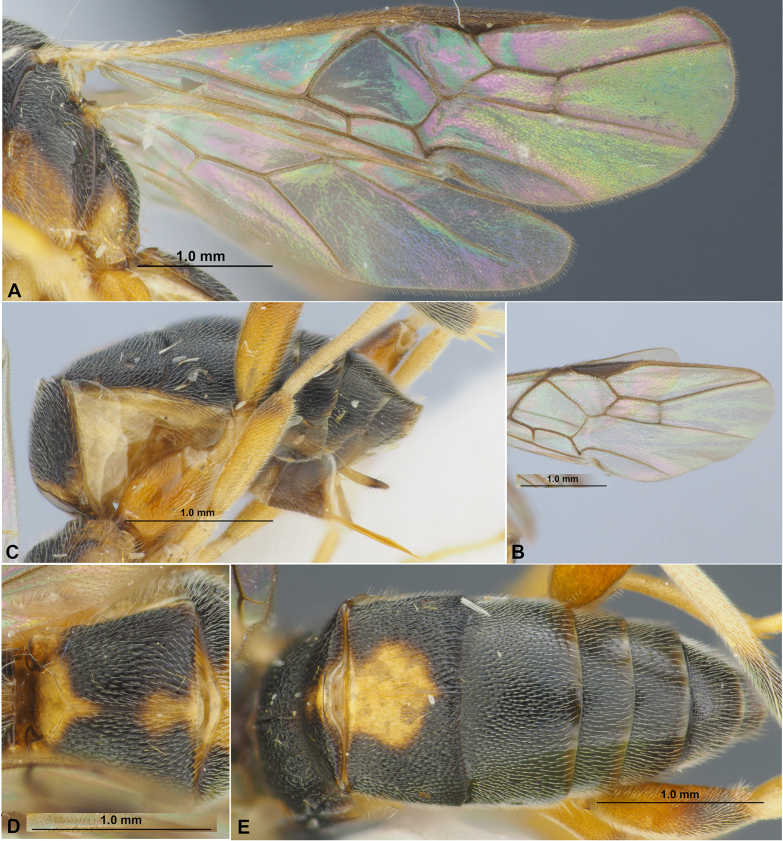
Aleiodes (Chelonorhogas) pseudalbitibia sp. nov., female, holotype. **A**. Wings; **B**. Apical half of fore wing; **C**. Metasoma, lateral view; **D**. First tergite of metasoma, dorsal view; **E**. Metasoma, dorsal view.

##### Description.

**Female. *Body*** length 6.3–6.7 mm; fore wing length 4.3–4.6 mm.

***Head*** width (dorsal view) 1.8–2.0× its median length, 1.1× width of mesoscutum. Occipital carina complete and distinct dorsally. Head behind eye (dorsal view) distinctly and weakly-curvedly narrowed; transverse diameter of eye 1.8–2.2× length of temple. Ocelli weakly enlarged, anterior ocellus small, ocelli arranged in almost equilateral triangle; POL 0.7–0.8× Od, 0.5–0.6× OOL; Od ~ 0.8× OOL. Eye rather high, weakly bean-shaped, 1.4–1.5× higher than broad (lateral view). Face weakly convex, its width 1.3–1.4× height of face and clypeus combined, 0.9× height of eye. Hypoclypeal depression small, circular, its width 0.7–0.8× distance from edge of depression to eye, 0.3× minimum width of face. Malar space 0.3–0.4× height of eye, 1.0–1.2× basal width of mandible. Head below eyes (front view) distinctly and weakly-curvedly narrowed. ***Antennae*** setiform, 48–51-segmented. Scape relatively long, 1.7–1.8× longer than its maximum width. First flagellar segment 1.4–1.7× longer than its apical width, 1.1–1.2× longer than second segment. Medial segments subsquadrate. Penultimate segment 1.3–1.5× longer than wide. Apical segment acuminated and with very short spine.

***Mesosoma*** 1.5–1.6× longer than high. Mesoscutum 1.1× wider than its medial length (dorsal view), highly and curvedly elevated above pronotum (lateral view). Prescutellar depression (scutal sulcus) relatively short, weakly curved, ~ 0.2× as long as scutellum, with three high carinae, weakly rugulose between carinae. Precoxal sulcus indistinct. Metanotum with low, wide, and obtuse dorsal tooth (lateral view). Propodeum convexly curved posteriorly, without lateral tubercles.

***Wings***. Fore wing 3.3–3.5× longer than its maximum width. Radial vein (r) arising weakly before middle of pterostigma. Radial (marginal) cell not shortened; metacarp (1-R1) 1.4–1.5× longer than pterostigma. First radial abscissa (r) 1.3–1.5× longer than maximum width of pterostigma. Second radial abscissa (3-SR) 1.7–2.0× longer than first abscissa (r) and formed obtuse angle with it, ~ 0.4× as long as third radial abscissa (SR1), 1.5–2.0× longer than first radiomedial vein (2-SR). Second radiomedial (submarginal) cell medium-length, 2.0–2.3× longer than its maximum width, 0.9–1.1× as long as brachial (subdiscal) cell. Recurrent vein (m-cu) straight, 1.6–1.8× longer than second abscissa of medial vein (2-SR+M). Nervulus (cu-a) straight, weakly oblique, distinctly postfurcal, distance (1-CU1) between basal vein (1-M) and nervulus (cu-a) 2.6–3.0× longer than nervulus (cu-a); first abscissa of cubital vein (1-CU1) ~ 0.7× its second abscissa (2-CU1). In hind wing, radial (marginal) cell distinctly widened distally. Radial vein (SR) arising from costal vein (2-SC+R) closely to basal vein (r-m). Nervulus (cu-a) almost straight or weakly curved. Recurrent vein (m-cu) completely absent.

***Legs***. Hind trochantellus 1.5–1.6× longer than hind trochanter (measured on their lower margins). Hind femur 3.4–3.5× longer than wide. Longest inner spur of hind tibia 0.45× as long as hind basitarsus. Hind tarsus 0.8× as long as hind tibia. Hind basitarsus 0.8–0.9× as long as second to fifth segments combined. Second segment of hind tarsus 0.3–0.4× as long as basitarsus, 1.0–1.1× as long as fifth segment (without pretarsus). Claw simple, not pectinate.

***Metasoma***. Tergites behind fourth tergite of metasoma distinctly protruding. First tergite distinctly and mostly almost evenly linearly widened towards apex, strongly widened just basally; length of tergite 0.9–1.0× its posterior width; posterior width 2.0–2.5× its anterior (basal) width. Basal area of first tergite rather wide and elongate, with three pointed break medio-posteriorly. Medial length of second tergite ~ 0.7× its basal width; 1.1–1.2× length of third tergite; its basal area short, but distinct. Suture between second and third tergites shallow, almost straight and crenulate. Ovipositor sheath 0.6–0.7× as long as hind basitarsus, 0.4–0.5× as long as first tergite.

***Sculpture and pubescence***. Vertex coarsely transversely and undulating striate with dense rugosity between striae; frons coarsely curvedly striate; temple entirely coarsely rugose-striate with fine additional granulation; face entirely densely rugose-reticulate with dense oblique striae. Mesoscutum and scutellum entirely densely rugulose-reticulate with small and very dense granulation, mesoscutum with rugosity on small medio-posterior area in posterior third. Mesopleuron sparsely finely punctate, space between punctulae smooth. Propodeum entirely distinctly rugulose-reticulate, with distinct and complete medial carina. First and second metasomal tergites entirely distinctly and densely striate with dense rugulosity and punctation, their basal areas smooth, present distinct and complete medial carinae; third tergite densely reticulate-punctate, with striation in basal third, without medial carina. Following tergites densely and finely punctate.

***Colour***. Body mostly black; prothorax anteriorly, mesopleuron in lower half and metapleuron mostly pale reddish brown to yellowish brown; spots on first tergite anteriorly (large) and sometimes medio-posteriorly (small) and on second tergite medially (large or small) yellow or brownish yellow; metasoma ventrally yellow or pale reddish brown mostly. Antennae entirely black. Palpi creamy yellow, infuscate basally. Fore and middle legs yellow with femora brownish yellow and middle femur faintly infuscate distally. Hind legs pale reddish brown or pale brown in basal half with distinctly infuscate femur in distal fifth to third; trochanter and trochantellus yellow or pale yellow, hind tibia in basal half and hind tarsus yellowish white, hind tibia black in distal half. Wing very faintly infuscate; pterostigma entirely black.

**Male**. Body length 6.0 mm; fore wing length 4.3 mm. Antennae 47-segmented. Otherwise similar to female.

##### Comparative diagnosis.

This new species is similar to the Palaearctic Aleiodes (Ch.) apicalis (Brulle, 1832) (*A. ductor* auct.) (van Achterberg et al. 2020), but very distinctly differs from it in having the second radiomedial (submarginal) cell rather long (short in *A. apicalis*), radial (marginal) cell of the fore wing not or only weakly shortened (distinctly shortened in *A. apicalis*), claws simple (not pectinate) (distinctly pectinate in *A. apicalis*), ocelli large (small in *A. apicalis*), palps pale yellow (brown to dark brown in *A. apicalis*), the metasoma mostly black and sometimes with yellowish spots on the base of first and middle of second tergites (first and second tergites entirely and basal half of third tergite pale reddish brown, apically black in *A. apicalis*), all coxae pale yellow (black to reddish brown partly in *A. apicalis*), hind tibia creamy yellow in its basal half and black in the apical half (entirely or mostly black in *A. apicalis*).

##### Etymology.

Named from a combination of the words: *pseudo* (Greek for “false”) and the species name *albitibia*, because this species is superficially similar to Aleiodes (A.) albitibia Wesmael.

##### Host.

Unknown.

##### Distribution.

Korean Peninsula.

##### Remarks.

This new species resembles superficially the Palaearctic Aleiodes (Aleiodes) albitibia Wesmael, 1838 from the subgenus *Aleiodes*, but differs from it, besides subgeneric character [widened posteriorly radial (marginal) cell of the hind wing], in the following characters: distinctly concave occiput in dorsal view (vs weakly concave), mostly black malar space of the head (vs mostly cream-white), small ocelli (vs enlarged), subvertical position of nervulus (cu-a) of the fore wing (vs distinctly oblique), cream-white pronotum dorsally (vs black or dark brown), and mostly pale hind tarsus (vs mostly darkened).

#### Aleiodes (Chelonorhogas) rufoniger

Taxon classificationAnimaliaHymenopteraBraconidae

Belokobylskij & Ku sp. nov.

E8703AB9-9CA3-5F42-8F8D-7D9E84E4774D

https://zoobank.org/E8B8FE2D-3E02-4A41-8597-A62D54080CEB

Figs 5, 6

##### Type material.

***Holotype*** • female, South Korea, [GN] Mt. Jiri, Naedae-ri, Sicheon-myeon, Sancheong-gun, Gyeongsangnam-do, Habudam, Mercury Lamp, 10–11.V.1997 (Jesik Jeon leg.) (NIBR). ***Paratypes***: South Korea [GG] • Kyounggi, Suweon, Mt. Kwanggyo, LT, 16.IV.1998 (Deok-Seo Ku), 2 males (SMNE, ZISP). **[GN]** • Gyeongsangnam-do, Gyoryong-ri, Geumosan, Geumseongsa, Jingyo-myeon, Hadong-gun, LT, 17–18.IV.1999 (Je-Sik Jean), 1 male (SMNE).

**Figure 5.**
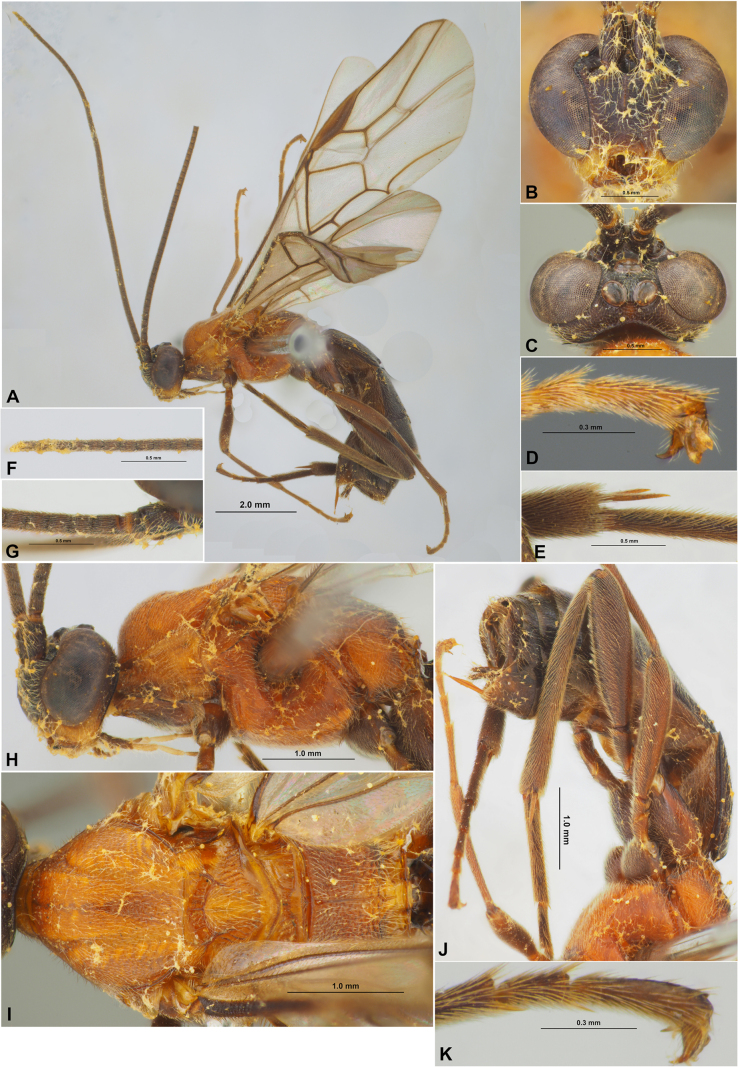
Aleiodes (Chelonorhogas) rufoniger sp. nov., female, holotype. **A**. Habitus, lateral view; **B**. Head, front view; **C**. Head, dorsal view; **D**. Claw of fore leg; **E**. Tibial spurs and basitarsus of hind leg, inner side; **F**. Apical segments of antenna; **G**. Basal segments of antenna; **H**. Head and mesosoma, lateral view; **I**. Mesosoma, dorsal view; **J**. Hind leg; **K**. Claw of hind leg.

**Figure 6.**
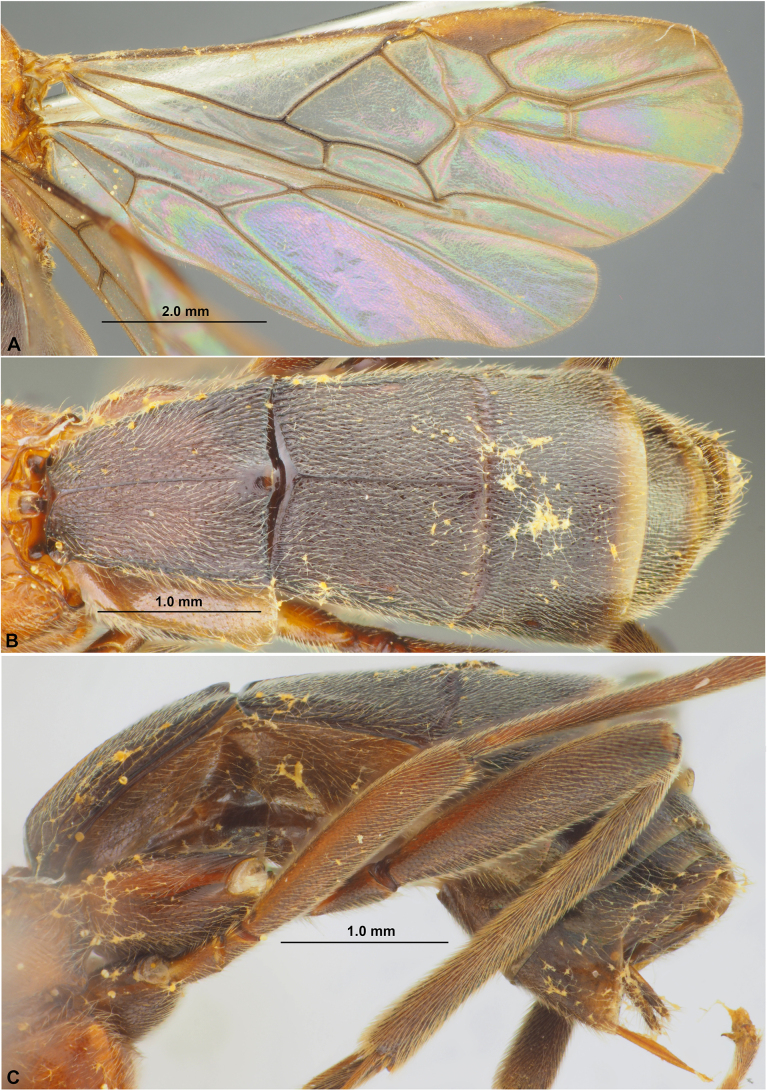
Aleiodes (Chelonorhogas) rufoniger sp. nov., female, holotype; **A**. Wings; **B**. Metasoma, dorsal view; **C**. Metasoma, lateral view.

##### Description.

**Female. *Body*** length 9.5 mm; fore wing length 8.6 mm.

***Head*** width (dorsal view) 2.1× its median length, 1.05× width of mesoscutum. Occipital carina complete and distinct dorsally. Head behind eye (dorsal view) strongly and almost linearly narrowed; transverse diameter of eye 2.8× length of temple. Ocelli strongly enlarged, anterior ocellus large, ocelli arranged in almost equilateral triangle; POL 0.3× Od, 2.0× OOL; Od ~ 7.0× OOL. Eye high, weakly bean-shaped, 1.6× higher than broad (lateral view). Face weakly convex, its width 0.8–0.9× height of face and clypeus combined, 0.5× height of eye. Hypoclypeal depression medium-sized, circular, its width 1.5× distance from edge of depression to eye, 0.5× minimum width of face. Malar space 0.15× height of eye, 0.5× basal width of mandible. Head below eyes (front view) strongly and weakly-curvedly narrowed. ***Antennae*** slender, weakly setiform, 67-segmented. Scape relatively long, ~ 2.0× longer than its maximum width. First flagellar segment 1.8× longer than its apical width, 1.2× longer than second segment. Medial segments weakly elongate. Penultimate segment 2.3× longer than wide. Apical segment with distinct and rather long ‘spine’.

***Mesosoma*** 1.8× longer than high. Mesoscutum approx. as wide as its medial length (dorsal view), highly and curvedly elevated above pronotum (lateral view). Prescutellar depression (scutal sulcus) relatively long, weakly curved posteriorly, ~ 0.3× as long as scutellum, with six or seven low and short carinae, weakly rugulose between carinae. Scutellum with short lateral carinae in basal third. Precoxal sulcus indistinct. Metanotum with low and obtuse dorsal tubercle (lateral view). Propodeum evenly convexly curved posteriorly, without lateral tubercles.

***Wings***. Fore wing 3.0× longer than its maximum width. Radial vein (r) arising weakly before middle of pterostigma. Radial (marginal) cell not shortened; metacarp (1-R1) 1.3× longer than pterostigma. First radial abscissa (r) approx. equal to maximum width of pterostigma. Second radial abscissa (3-SR) 1.7× longer than first abscissa (r) and formed very obtuse angle with it, ~ 0.4× as long as third radial abscissa (SR1), 1.7× longer than first radiomedial vein (2-SR). Second radiomedial (submarginal) cell medium-length, 2.6× longer than its maximum width, almost as long as brachial (subdiscal) cell. Recurrent vein (m-cu) straight, 2.2× longer than second abscissa of medial vein (2-SR+M). Nervulus (cu-a) straight, weakly oblique, distinctly postfurcal, distance (1-CU1) between basal vein (1-M) and nervulus (cu-a) almost 2.0× longer than nervulus (cu-a); first abscissa of cubital vein (1-CU1) 0.5× its second abscissa (2-CU1). In hind wing, radial (marginal) cell distinctly widened distally. Radial vein (SR) arising from costal vein (2-SC+R) not far from basal vein (r-m). Nervulus (cu-a) weakly curved. First abscissa of mediocubital vein (M+CU) 1.3× longer than second abscissa (1M). Recurrent vein (m-cu) completely absent.

***Legs***. Hind trochantellus ~ 2.0× longer than hind trochanter (measured on their lower margins). Hind femur 4.3× longer than wide. Longest inner spur of hind tibia 0.35× as long as hind basitarsus. Inner distal margin of hind tibia without comb of dense pale setae. Hind tarsus approx. as long as hind tibia. Hind basitarsus 0.8× as long as second to fifth segments combined. Second segment of hind tarsus 0.4× as long as basitarsus, 1.3× as long as fifth segment (without pretarsus). Claw simple, not pectinate.

***Metasoma***. Segments behind fourth tergite distinctly protruding. First tergite distinctly and almost evenly linearly widened towards apex, not strongly widened basally; length of tergite 1.2× its posterior width; posterior width 2.7× its anterior (basal) width. Basal area of first tergite rather wide and elongate, with singly pointed medial break posteriorly. Medial length of second tergite approx. equal to its anterior width, 1.3× length of third tergite; its smooth basal area short, but distinct. Suture between second and third tergites shallow, weakly curved and crenulate. Ovipositor sheath 0.3× as long as hind basitarsus, 0.25× as long as first tergite.

***Sculpture and pubescence***. Vertex distinctly transversely curvedly striate with reticulation and granulation between striae; frons without rugosity, coriaceous to smooth; temple coarsely transversely striate with fine rugulosity between striae; face densely transverse striate with dense rugulosity between striae. Mesoscutum entirely densely granulate with rugosity on area in medio-posterior third. Scutellum granulate-reticulate. Mesopleuron rather finely punctate, space between punctulae smooth; anteriorly almost entirely smooth. Propodeum entirely distinctly rugulose-reticulate, with distinct and complete medial carina. First and second metasomal tergites entirely finely and densely striate with dense rugulosity, their basal areas smooth, with distinct and complete medial carinae; third tergite densely reticulate-coriaceous, with dense fine striation in basal half, without medial carina. Following tergites densely and finely punctate-coriaceous.

***Colour***. Head mostly black, malar space and lower of face yellow. Mesosoma entirely pale reddish brown. Metasoma entirely black. Antennae entirely black. Palpi dark brown basally and yellow apically. Legs dark reddish brown, partly almost black, hind tibia in basal half dark. Wing faintly infuscate; pterostigma mostly dark brown, paler basally, laterally, and apically.

**Male**. Body length 7.7–8.9 mm; fore wing length 6.7–7.0 mm. Antennae 62–64-segmented. Medial length of second tergite 0.9–1.0× its basal width, 1.2–1.3× length of third tergite. Third tergite with medial carina at least in basal half. Palpi mostly dark reddish brown to almost black. Legs mostly black. Otherwise similar to female.

##### Comparative diagnosis.

This new species is very similar to Aleiodes (Chelonorhogas) daisetsuzanus (Watanabe, 1937) from the Eastern Palaearctic (Watanabe 1937), but differs by having the tarsal claws not pectinate (distinctly pectinate because of the long and pale pectens in *A. daisetsuzanus*), metasoma entirely black with first and second tergites distinctly densely striate (mostly reddish brown with first and second tergites finely punctate with reticulation in *A. daisetsuzanus*), maxillary palps dark brown basally and yellow apically (entirely yellow in *A. daisetsuzanus*), and the hind tibia entirely black (pale brown or yellow in basal half and dark to black in apical half in *A. daisetsuzanus*).

##### Etymology.

Named from a combination of the words: *rufus* (Latin for rufous, reddish-brown) and *niger* (Latin for black), referring to the presence of both colours on the body of the new species.

##### Host.

Unknown.

##### Distribution.

Korean Peninsula.

##### Remarks.

*Aleiodes rufoniger* sp. nov. resembles superficially resembles the species from the subgenus *Arcaleiodes*, namely A. (Arc.) nitidus Chen & He, 1991 and A. (Arc.) hubeiensis from China (Chen and He 1991, 1997), but differs from them by having the metasoma mostly black and only the first tergite partly dark reddish brown and without a pale band (vs first and second metasomal tergites entirely or partly reddish yellow and sometimes first tergite with pale band), tibia of the hind legs entirely dark reddish brown to black (vs entirely reddish yellow or brown and basally with pale area), and three apical segments of the maxillary palp yellow and three basal segments brown (vs maxillary palp brown entirely or largely so).

#### Aleiodes (Aleiodes) crassicornis

Taxon classificationAnimaliaHymenopteraBraconidae

Belokobylskij & Ku sp. nov.

C42FBC6A-C50F-5E70-BD2B-6999571D2BEB

https://zoobank.org/9D2DCFC0-4068-4ECD-8009-A71041EEBC0C

Figs 7, 8

##### Type material.

***Holotype*** • female, South Korea, “Korea [GN], Sanglakwon, Panmun-dong, Jiju-si, 28.VI.2023, Gwanseok Lee (light trap)” (NIBR). ***Paratypes*: [CB]** • Cheondong-ri, Cheondong Valley, Sobaeksan, Danyang-eup, Danyang-gun, 14.VIII.1998 (Jeon Jesik), 1 female (SMNE). **[GB]** • Andong, Pukhu, Shinjeon, Mt. Hakga, LT, 19.VII.1998 (Deok-Seo Ku), 1 female (ZISP). **[GW]** • Gangreung-si, MT, 11–25.VI.2018 (Hyung-Keun Lee), 1 female (ZISP). **[JB]** • Myeongdeok-ri, Janggye-myeon, Jangsu-gun, Jeollabuk-do, Yuksip-ryeong Rest Area, LT, 15.VII.2016 (Deokseo Ku, Tae-Ho An, Hye-Rin Lee), 1 female (SMNE).

**Figure 7.**
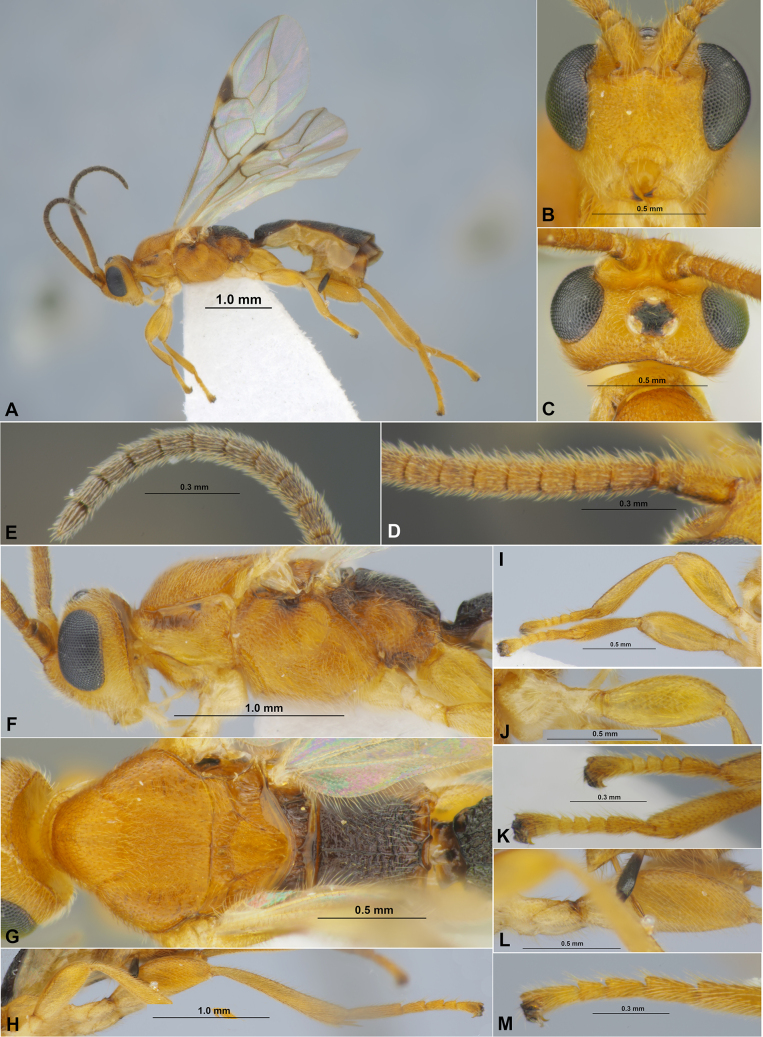
Aleiodes (Aleiodes) crassicornis sp. nov., female, holotype. **A**. Habitus, lateral view; **B**. Head, front view; **C**. Head, dorsal view; **D**. Basal segments of antenna; **E**. Apical segments of antenna; **F**. Head and mesosoma, lateral view; **G**. Mesosoma, dorsal view; **H**. Hind leg; **I**. Fore legs; **J**. Fore coxa and femur; **K**. Tarsus and claw of fore leg; **L**. Hind femur; **M**. Hind tarsus and claw.

**Figure 8.**
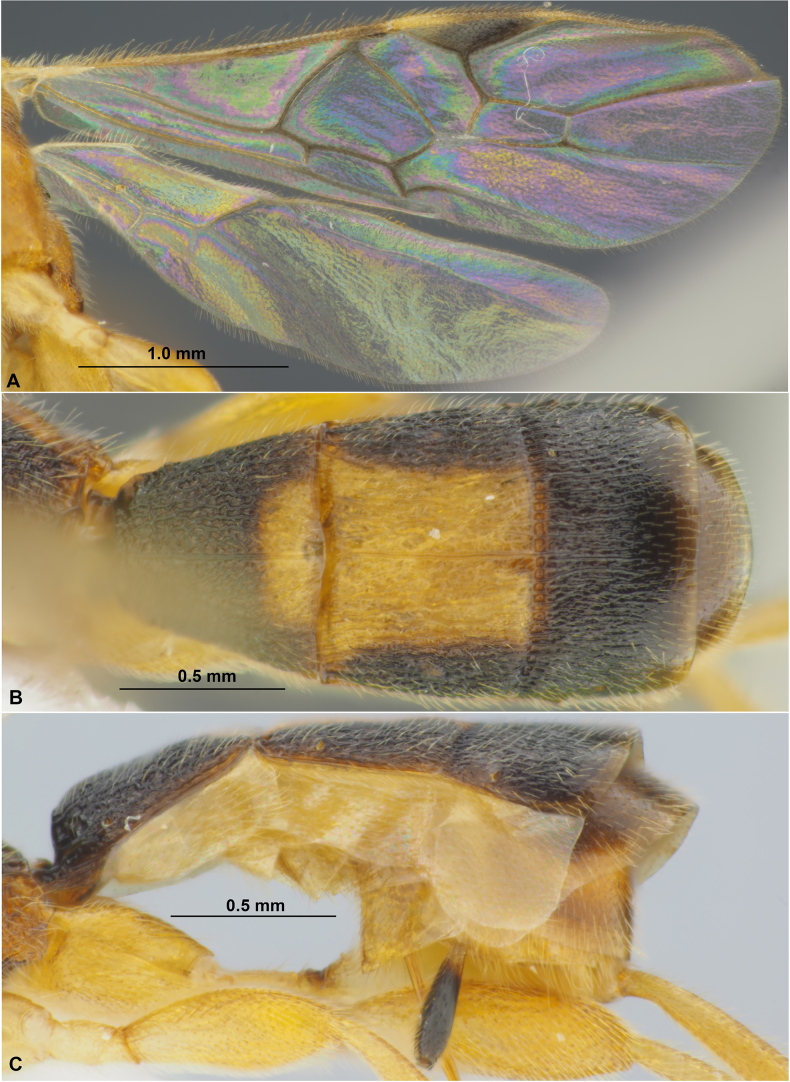
Aleiodes (Aleiodes) crassicornis sp. nov., female, holotype. **A**. Wings; **B**. Metasoma, dorsal view; **C**. Metasoma, lateral view.

##### Description.

**Female. *Body*** length 4.0–4.9 mm; fore wing length 3.0–3.8 mm.

***Head*** width (dorsal view) 1.8–2.0× its median length, 1.0–1.1× width of mesoscutum. Occipital carina complete and rather distinct mediodorsally. Head behind eye (dorsal view) curvedly narrowed; transverse diameter of eye 2.0–2.2× length of temple. Ocelli weakly enlarged, arranged in triangle with base ~ 1.2× its sides; POL almost equal to Od, 0.6–0.7× OOL; Od 0.6–0.7× OOL. Eye rather high, weakly bean-shaped, 1.6–1.7× higher than broad (lateral view). Face distinctly convex, its width 1.2–1.3× height of face and clypeus combined, 0.9–1.0× height of eye. Hypoclypeal depression small, circular, its width 0.6–0.7× distance from edge of depression to eye, ~ 0.3× minimum width of face. Malar space ~ 0.4× height of eye, 1.1–1.3× basal width of mandible. Head below eyes (front view) distinctly and weakly-curvedly narrowed. ***Antennae*** thick, shortened, weakly setiform, 23–25-segmented, almost as long as head and mesosoma combined. Scape relatively long, widened distally, ~ 1.5× longer than its maximum width. First flagellar segment 1.4–1.5× longer than its apical width, ~ 1.1× longer than second segment. Medial flagellar segments weakly elongated. Penultimate segment 1.5–1.8× longer than wide, 0.7–0.9× as long a first flagellar segment. Apical segment acuminate and with short spine.

***Mesosoma*** 2.2–2.3× longer than high. Neck of prothorax very short. Mesoscutum 0.9–1.0× as wide as its medial length (dorsal view), distinctly and curvedly elevated above pronotum (lateral view). Prescutellar depression (scutal sulcus) relatively short, weakly curved, ~ 0.3× as long as scutellum, with 6–8 high carinae, weakly rugulose to almost smooth between carinae. Precoxal sulcus present, but very shallow, densely rugulose. Metanotum without dorsal tooth (lateral view). Propodeum weakly curvedly oblique in posterior half (lateral view), without lateral tubercles.

***Wings***. Fore wing 3.2–3.4× longer than its maximum width. Radial vein (r) arising weakly before middle of pterostigma. Radial (marginal) cell not shortened; metacarp (1-R1) 1.3–1.5× longer than pterostigma. First radial abscissa (r) weakly curved, 1.0–1.3× as long as maximum width of pterostigma. Second radial abscissa (3-SR) 1.5–1.7× longer than first abscissa (r) and formed obtuse angle with it, ~ 0.4× as long as third radial abscissa (SR1), 1.7–2.1× longer than first radiomedial vein (2-SR). Second radiomedial (submarginal) cell medium-length, relatively narrow, 2.3–2.4× longer than its maximum width, approx. as long as brachial (subdiscal) cell. Second medial abscissa (2-SR+M) 0.7× first radial abscissa (r). Recurrent vein (m-cu) almost straight, 1.6–2.0× longer than second abscissa of medial vein (2-SR+M), 1.2–1.3× first radiomedial vein (2-SR). Nervulus (cu-a) straight, subperpendicular, distinctly postfurcal, distance (1-CU1) between basal vein (1-M) and nervulus (cu-a) 1.4–1.6× longer than nervulus (cu-a); first abscissa of cubital vein (1-CU1) ~ 0.4× its second abscissa (2-CU1). In hind wing, radial (marginal) cell subparallel-sided in basal half and weakly widened distally. Radial vein (SR) arising from costal vein (2-SC+R) far from basal vein (r-m). Nervulus (cu-a) weakly curved. First abscissa of mediocubital vein (M+CU) 2.1× longer than second abscissa (1-M). Recurrent vein (m-cu) present, short, strongly unsclerotised, antefurcal.

***Legs***. All legs thickened. Fore femur 2.3–2.4× longer than its maximum width; fore and middle tibiae distinctly widened distally; segments of fore and middle tarsi shortened; middle femur ~ 2.7× longer than its maximum width. Hind trochantellus 1.5–1.6× longer than hind trochanter (measured on their lower margins). Hind femur very wide, 2.5–2.7× longer than its maximum width. Hind tibia weakly widened posteriorly, without comb of dense pale setae on its inner posterior margin. Longest inner spur of hind tibia ~ 0.5× as long as hind basitarsus. Hind tarsus 0.9–1.0× as long as hind tibia; segments of hind tarsus long and weakly thickened. Hind basitarsus 0.6–0.7× as long as second to fifth segments combined. Second segment of hind tarsus 0.4× as long as basitarsus, approx. as long as fifth segment (without pretarsus). Claw simple, not pectinate, but at least with one distinct bristle situated deeply basally.

***Metasoma***. Tergites behind third one weakly protruding. First tergite distinctly and mostly almost evenly linearly widened towards posterior margin, more distinctly widened just basally; length of tergite almost equal to its posterior width; posterior width 2.7–2.8× its anterior (basal) width. Basal area of first tergite wide and short, semi-round, with single pointed break medio-posteriorly. Medial length of second tergite 0.8× its anterior width; 1.2–1.3× length of third tergite; its basal area short and indistinct. First and second tergites with complete and third tergite in anterior half with distinct carina. Suture between second and third tergites distinct, weakly curved and coarsely crenulate. Ovipositor sheath ~ 0.8× as long as hind basitarsus, ~ 0.5× as long as first tergite.

***Sculpture and pubescence***. Vertex and temple densely reticulate-rugose with fine granulation; frons mostly finely or distinctly granulate, rugulose laterally; face transversely undulating striate with reticulation, partly granulate. Mesoscutum and scutellum entirely densely and finely reticulate-coriaceous, mesoscutum densely longitudinally striate in medio-posterior half. Mesopleuron mostly finely reticulate-rugulose with curved striation, coriaceous partly, its posterior and upper wide areas (speculum) smooth. Propodeum entirely distinctly rugulose-reticulate with striation partly; with distinct and complete medial carina. Hind coxa curvedly aciculate dorsally, densely and finely granulate-reticulate laterally; hind femur densely and finely granulate with sparse punctation. First to third metasomal tergites entirely distinctly and densely striate with dense rugulosity, third tergite finely sculptured posteriorly; anterior triangular area of first tergite almost smooth; first and second tergites with complete medial carinae, carina of third tergite distinct in basal half only. Following tergites densely and finely or very finely coriaceous.

***Colour***. Body mostly yellow to brownish yellow; propodeum and first to third tergites mostly dark reddish brown to almost black, first tergite medio-posteriorly and second tergite widely medially with brownish yellow spots; tergite behind third one reddish brown. Antennae yellow to brownish yellow or pale reddish brown in basal half, brown, reddish brown to almost black in posterior half. Palpi creamy yellow. Legs mostly yellow to brownish yellow, basal parts of fore and middle legs pale yellow. Wing subhyaline; pterostigma medially widely brown, pale yellow in basal third and apical fifth.

**Male**. Unknown.

##### Comparative diagnosis.

This new species is similar to Aleiodes (A.) tsukubaensis Belokobylskij, 2000 from Japan (Belokobylskij 2000), but differs from it by having the antenna thick and short (thinner and longer in *A. tsukubaensis*), basal segments of antenna short (long in *A. tsukubaensis*), legs strongly widened, fore femur 2.3–2.4× and middle femur 2.5–2.7× longer than their maximum width, respectively (less widened, fore femur 3.3× and middle femur 3.0× longer than their maximum width in *A. tsukubaensis*).

This new species is similar to *A. globofemurus* Quicke & Butcher, 2012 described from Thailand (Butcher et al. 2012: spelled correctly on the pages 4, 6 and 106, but erroneously as A.*globifemurus* on the pages 19 and 107), but differs from it by having the occipital carina complete and rather distinct mediodorsally (obliterated mediodorsally in *A. globofemurus*), antenna 23–25-segmented (31-segmented in *A. globofemurus*), second medial abscissa (2-SR+M) of the fore wing 0.7× as long as the first radial abscissa (r) (1.1× in *A. globofemurus*), radial (marginal) cell of the hind wing subparallel-sided in basal half and weakly widened distally (entirely more or less parallel in *A. globofemurus*), first abscissa of mediocubital vein (M+CU) of the hind wing 2.1× longer than the second abscissa (1-M) (1.75× in *A. globofemurus*), claw simple, not pectinate, but at least with one distinct bristle situated deep basally (claw with pectin of 3 or 4 small teeth from the base to the middle in *A. globofemurus*), mesopleuron mostly finely reticulate-rugulose with curved striation, coriaceous partly, its posterior and upper wide areas (speculum) smooth (almost entirely aciculate in *A. globofemurus*), carina of third tergite distinct in the basal half (weakly differentiated on anterior 0.2 in *A. globofemurus*), malar space entirely yellow (cream-yellow in *A. globofemurus*), and propodeum and most part of metasoma brown, dark brown to black (entirely pale reddish brown to yellowish brown in *A. globofemurus*).

##### Etymology.

This species is named after *crassus* (Latin for thick) and *cornis* (Latin for horn or projection), referring to its thick antenna.

##### Host.

Unknown.

##### Distribution.

Korean Peninsula.

#### Aleiodes (Aleiodes) heterogamoides

Taxon classificationAnimaliaHymenopteraBraconidae

Belokobylskij & Ku sp. nov.

8457D042-E05B-5AAF-AB36-256A5659E49D

https://zoobank.org/D2F1FE13-009D-4AD6-BC33-72C4F600AB77

Figs 9, 10

##### Type material.

***Holotype*** • female, South Korea, “Korea (JJ), Mulyeongari, Sumang-ri, Namwon-up, Seoqwipo-si, Jeju-do, VII.07–VII.30.2017 (Malaise trap)” (collector unknown) (NIBR). ***Paratypes***: South Korea: **[GG]** • Kyeonggi-do, Suwon-shi, Seodun-dong, Mt. Yeogi, MT, 23–29.VI.1994 (Deokseo Ku), 1 male (SMNE); same locality, 29.VI–6.VII.1994, 1 male (ZISP). **[GN]** • Beoppyeong-ri, Chahwang-myeon, Sancheong-gun, 35.48604 N, 127.9746 E, 22.VII.2024 (V. Chemyreva), 3 females (SMNE, ZISP). **[JJ]** • Eoseungsaengak, Haeando, Jeju-si, Jeju-do, MT, 14–29.X.2017 (collector unknown), 5 females (SMNE, ZISP) • same label, but 3–16.IX.2017, 2 males (SMNE, ZISP) • same label, but 6–19.VIII.2017, 2 males (SMNE, ZISP) • same label, but 19.VIII–3.IX.2017, 1 male (SMNE) • same label, but 16–30.IX.2017, 1 female (SMNE); same label as in holotype, 1 male (ZISP) • same label as in holotype, but 12–26.VIII.2017, 1 male (SMNE) • Manse-hill, Gwangryeong-ri, Aewol-up, Jeju-si, Jeju-do, MT, 12–21.VIII.2017 (collector unknown), 1 female (SMNE) • Gyorae Natural Recreation Forest, Gyorae-ri, Jocheon-eup, Jeju-si, MT № 11, 15–31.VIII.2023 (Deokseo Ku, Muncheol Kwon), 4 females (SMNE, ZISP) • Jeonnam National University Academic Forest, 98-Ora 2dong, Jeju-si, MT № 11, 15–31.VIII.2023 (Deokseo Ku, Muncheol Kwon), 1 female (SMNE) • Jeolmul Natural Recreation Forest, San 78-1, a car shelter, Bonggae-dong, Jeju-si, forest, MT-A-№ 7, 15–30.IX.2023 (Deokseo Ku, Muncheol Kwon), 1 female, 3 males (SMNE, ZISP) • Georin deer Observatory, San 2-11, Daepo-dong, Seogwipo-si, MT-№ 7, 15–31.VIII.2023 (Deokseo Ku, Muncheol Kwon), 1 male (SMNE) • Mt. Sanbangsan, Sanbang-ro, 126-gil, Andeok-myeon, Seogwipo-si, MT-№ 8, 15–30.IX.2023 (Deokseo Ku, Muncheol Kwon), 1 male (SMNE) • Jeolmul Natural Recreation Forest, San 78-1, Bonggae-dong, Jeju-si, MT-A-№ 7, 1–15.VII.2024 (Deokseo Ku, Muncheol Kwon), 2 females (SMNE) • near the Manjanggul Cave, Woljeong-ru, Gujwa-eup, Jeju-si B, MT-4, 15–30.VII.2024 (Deokseo Ku, Muncheol Kwon), 3 females, 2 males (SMNE, ZISP) • Gyorae-ri, Jocheon-eup, Jeju-si, a car shelter, MT-№ 6, 1–15.VIII.2024 (Deokseo Ku, Muncheol Kwon), 1 male (SMNE).

**Figure 9.**
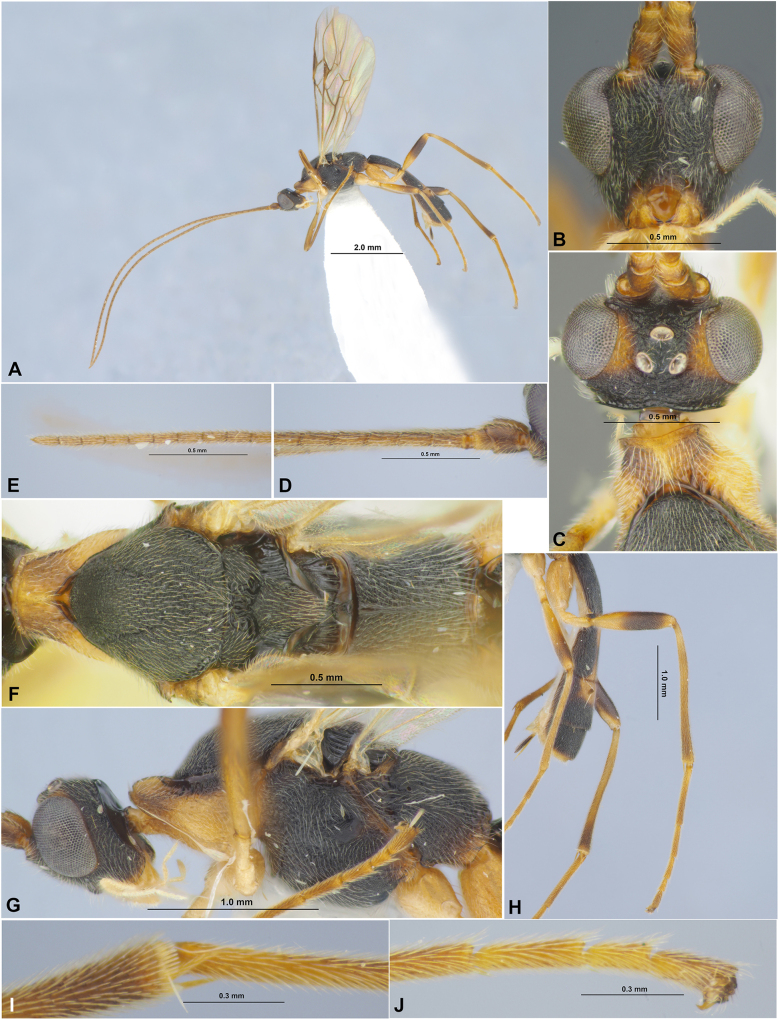
Aleiodes (Aleiodes) heterogamoides sp. nov., female, holotype. **A**. Habitus, lateral view; **B**. Head, front view; **C**. Head and anterior part of mesosoma, dorsal view; **D**. Basal segments of antenna; **E**. Apical segments of antenna; **F**. Mesosoma, dorsal view; **G**. Head and mesosoma, lateral view; **H**. Hind leg; **I**. Apex of tibia, spurs and basitarsus of hind leg, inner side; **J**. Claw of hind leg.

**Figure 10.**
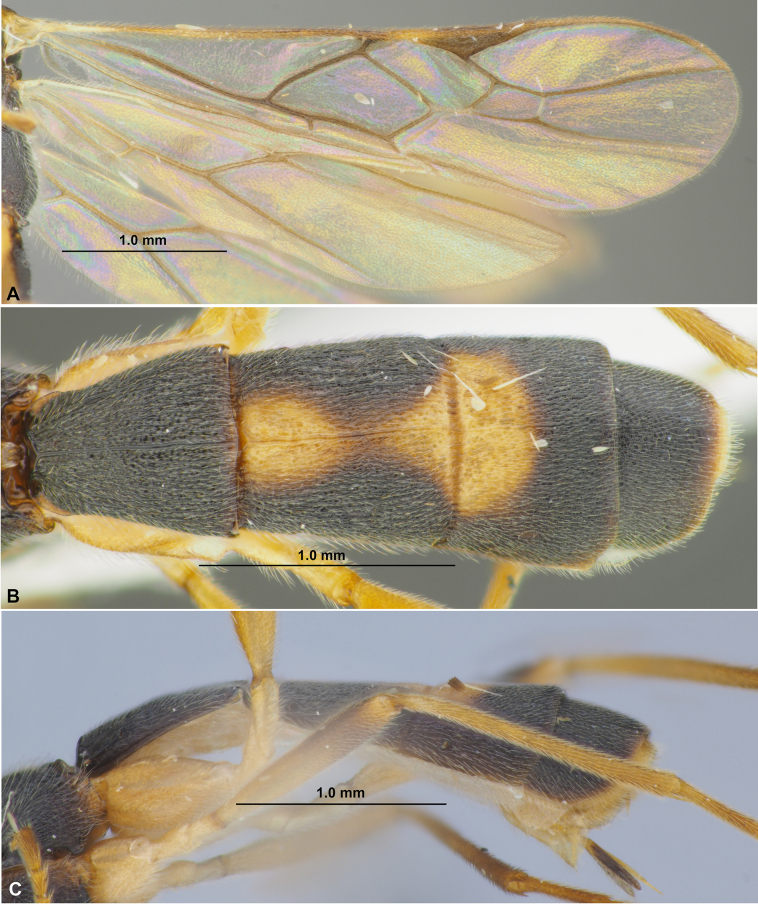
Aleiodes (Aleiodes) heterogamoides sp. nov., female, holotype. **A**. Wings; **B**. Metasoma, dorsal view; **C**. Metasoma, lateral view.

##### Description.

**Female. *Body*** length 5.2–5.8 mm; fore wing length 4.0–4.3 mm.

***Head*** width (dorsal view) 1.6–1.7× its median length, 1.15–1.20× width of mesoscutum. Occipital carina strong and complete dorsally. Head behind eye (dorsal view) strongly and almost linearly narrowed; transverse diameter of eye 2.3–2.4× length of temple. Ocelli weakly enlarged, arranged in almost equilateral triangle; POL 0.6–0.8× Od, 0.6–0.7× OOL; Od 0.9–1.0× OOL. Eye high, bean-shaped, 1.3–1.4× higher than broad (lateral view). Face weakly convex, its width 0.9–1.0× height of face and clypeus combined, 0.8–0.9× height of eye. Hypoclypeal depression small, circular, its width ~ 0.7× distance from edge of depression to eye, 0.30–0.35× minimum width of face. Malar space 0.3–0.4× height of eye, 1.0–1.3× basal width of mandible. Head below eyes (front view) distinctly and almost linearly narrowed. ***Antennae*** slender, weakly setiform, 50–52-segmented. Scape relatively long, 1.6–1.8× longer than its maximum width. First segment of flagellum 2.5–3.0× longer than its apical width, 1.1–1.2× longer than second segment. Penultimate segment 2.5–2.8× longer than wide. Apical segment acuminated and with short ‘spine’.

***Mesosoma*** 1.9–2.1× longer than high. Neck of prothorax long, almost straight anteriorly (dorsal view), dorsally with distinct longitudinal medial and upper rounded wide crest (roller). Mesoscutum 0.8–0.9× wider than its medial length (dorsal view), highly and curvedly elevated above pronotum (lateral view). Prescutellar depression (scutal sulcus) relatively long, ~ 0.3× as long as scutellum, with two-three high carinae, densely rugulose between carinae. Precoxal sulcus very shallow, rather wide, weakly oblique, densely rugose-reticulate. Metanotum with low or very low obtuse dorsal tooth (lateral view). Propodeum evenly curved posteriorly.

***Wings***. Fore wing 3.5–3.8× longer than its maximum width. Radial vein (r) arising from or weakly behind middle of pterostigma. Radial (marginal) cell not shortened; metacarp (1-R1) 1.2–1.5× longer than pterostigma. First radial abscissa (r) 1.3–2.0× longer than maximum width of pterostigma. Second radial abscissa (3-SR) 0.8–1.1× as long as first abscissa (r) and formed very obtuse angle with it, 0.2–0.3× as long as third radial abscissa (SR1), 1.1–1.2× longer than first radiomedial vein (2-SR). Second radiomedial (submarginal) cell short, distinctly narrowed distally, 1.9–2.0× longer than its maximum width, ~ 0.7× as long as relatively wide brachial (subdiscal) cell. Recurrent vein (m-cu) straight, 1.5–1.7× longer than second abscissa of medial vein (2-SR+M). Nervulus (cu-a) almost straight, weakly oblique, strongly postfurcal, distance (1-CU1) between basal vein (1-M) and nervulus (cu-a) 2.0–3.2× longer than nervulus (cu-a); first abscissa of cubital vein (1-CU1) 0.6–0.7× its second abscissa (2-CU1). In hind wing, radial (marginal) cell not or only weakly widened distally. Radial vein (SR) arising from costal vein (2-SC+R) closely to basal vein (r-m); third abscissa of costal vein (2-SC+R) short and thick, weakly elongate. First abscissa of mediocubital vein (M+CU) 1.4–1.6× longer than second abscissa (1-M) Nervulus (cu-a) distinctly evenly curved. Recurrent vein (m-cu) completely absent.

***Legs***. Hind trochantellus 1.5–1.7× longer than hind trochanter (measured on their lower margins). Hind femur 5.5–5.7× longer than wide. Longest inner spur of hind tibia 0.25–0.30× as long as hind basitarsus. Inner distal margin of hind tibia with comb of dense and long pale setae. Hind tarsus 1.0–1.1× as long as hind tibia. Hind basitarsus ~ 0.7× as long as second to fifth segments combined. Second segment of hind tarsus 0.50–0.55× as long as basitarsus, 1.8–2.0× longer than fifth segment (without pretarsus). Claw simple, without pectens or bristles.

***Metasoma***. Present four dorsally visible tergites. First tergite distinctly and almost evenly linearly widened towards apex, with small subbasal lateral lobes; length of tergite 1.2–1.3× its posterior width; posterior width 2.0–2.2× its anterior width. Basal area of first tergite rather wide and weakly elongated, with single pointed medio-posterior break. Second tergite elongate, its medial length 1.2–1.3× anterior width; ~ 1.3× length of third tergite; its basal area very short, almost invisible. Suture between second and third tergites deep, almost straight, crenulate. At least second to fourth tergites with lateral crease and separated laterotergites. Fourth tergite convex in posterior margin (dorsal view), without ventro-lateral notch (lateral view); fourth tergite 1.5–1.6× as long as third tergite. Segments behind fourth tergite usually mostly hidden under fourth one. Ovipositor sheath ~ 0.4× as long as hind basitarsus, 0.3–0.4× as long as first tergite.

***Sculpture and pubescence***. Vertex with coarse, undulate, transverse striate with dense rugosity between striae; frons coarsely rugose-reticulate partly with curved striae; temple entirely rugose-reticulate with granulation; face entirely densely granulate with small reticulation and usually with short oblique striae medially. Mesoscutum and scutellum entirely densely granulate-reticulate, mesoscutum with rugosity on narrow medio-posterior area. Mesopleuron almost entirely densely reticulate-rugose and partly with granulation, with small finely reticulate or smooth medio-posterior area. Propodeum entirely, densely, and distinctly rugulose-reticulate with small granulation, with medial carina in basal 0.5–0.7. First and second metasomal tergites entirely distinctly and densely striate with dense rugosity; third and fourth tergites entirely densely reticulate-rugulose with granulation. First and second tergites with complete medial carinae; third tergite with weak medial carina only in basal half.

***Colour***. Body mostly black; entirely or at most part (except dorsum) of prothorax cream-yellow, medial spots on centres of second (longitudinal) and third (basal transverse) tergites pale brown, yellow or pale yellow. Antennae pale reddish brown to (dark) reddish brown in basal half, brown to almost black in apical half. Palpi yellow. Legs basally brownish or pale yellow, femora at least in distal third or half brown to dark brown; all tibiae mostly brown to dark brown; all tarsi infuscate. Wing faintly infuscate; pterostigma brown, pale in basal quarter.

**Male**. Body length 5.5–6.0 mm; fore wing length 3.8–4.2 mm. Antenna 50–52-segmented. Second radiomedial (submarginal) cell of fore wing usually less distinctly narrowed distally. Otherwise similar to female.

##### Comparative diagnosis.

The new species is similar to *A. tashimai* (Kusigemati, 1983) from Japan and Korea (Kusigemati 1983), but differs from it by having the antenna without a pale ring of segments (with numerous pale segments in apical half of antenna in *A. tashimai*), the second abscissa of radial vein (3-SR) distinctly shorter than first abscissa (r) (with *Heterogamus* type of wing venation) (distinctly longer in *A. tashimai*), the second radiomedial (submarginal) cell short and usually distinctly narrowed distally (longer and weakly narrowed distally in *A. tashimai*), and the prothorax entirely or mostly pale yellow (entirely black in *A. tashimai*).

Additionally, the new species is similar to *A. narangae* (Rohwer, 1934) from eastern and south-eastern Asia (Chen and He 1997), but differ from it by having the pronotum dorsally with a distinct longitudinal crest (without crest in *A. narangae*), second abscissa of radial vein (3-SR) distinctly shorter than first abscissa (r) (distinctly longer in *A. narangae*), the second radiomedial (submarginal) cell short and usually distinctly narrowed distally (longer and weakly narrowed distally in *A. narangae*), prothorax entirely or mostly pale yellow (entirely black in *A. narangae*), and body mostly dark brown or black (entirely or mostly pale reddish brown in *A. narangae*).

In addition, *Aleiodes heterogamoides* sp. nov. is similar to *A. procarinatus* Quicke & Butcher, 2012 from Thailand (Butcher et al. 2012), but differs from it by having the medial longitudinal carina of propodeum present only in the basal half (virtually complete in *A. procarinatus*), submedial (subbasal) cell of the fore wing entirely setose (with glabrous line posteriorly in *A. procarinatus*), second abscissa of radial vein (3-SR) 0.8–1.1× first abscissa (r) (1.6× in *A. procarinatus*), inner distal margin of hind tibia with comb of dense long setae (without comb of setae in *A. procarinatus*), claw not pectinate (with inconspicuous pectin in base in *A. procarinatus*), antenna without ring of white segments (with a ring of 17 white segments in *A. procarinatus*), and prothorax mostly whitish yellow (mostly brown to dark brown in *A. procarinatus*).

##### Etymology.

Named from a combination of the genus name *Heterogamus* and the Latin suffix -*oides* (resembling) because the wing venation of the new species is very similar to that of the *Heterogamus* species.

##### Host.

Unknown.

##### Distribution.

Korean Peninsula.

#### Aleiodes (Aleiodes) imberbis

Taxon classificationAnimaliaHymenopteraBraconidae

Belokobylskij & Ku sp. nov.

8DB4C270-F581-54ED-9C94-E24F4862334A

https://zoobank.org/C251DBA8-FA33-4052-9376-84463646666E

Figs 11, 12

##### Type material.

***Holotype*** • female, South Korea, Gyeongsangnam-do (GN), Danjibong Peak (Kaya Mountain), Chiin-ri, Gaya-myeon, Hapcheon-gun, LT, 13–14.VII.1999 (Lee Hyun-ji) (NIBR).

**Figure 11.**
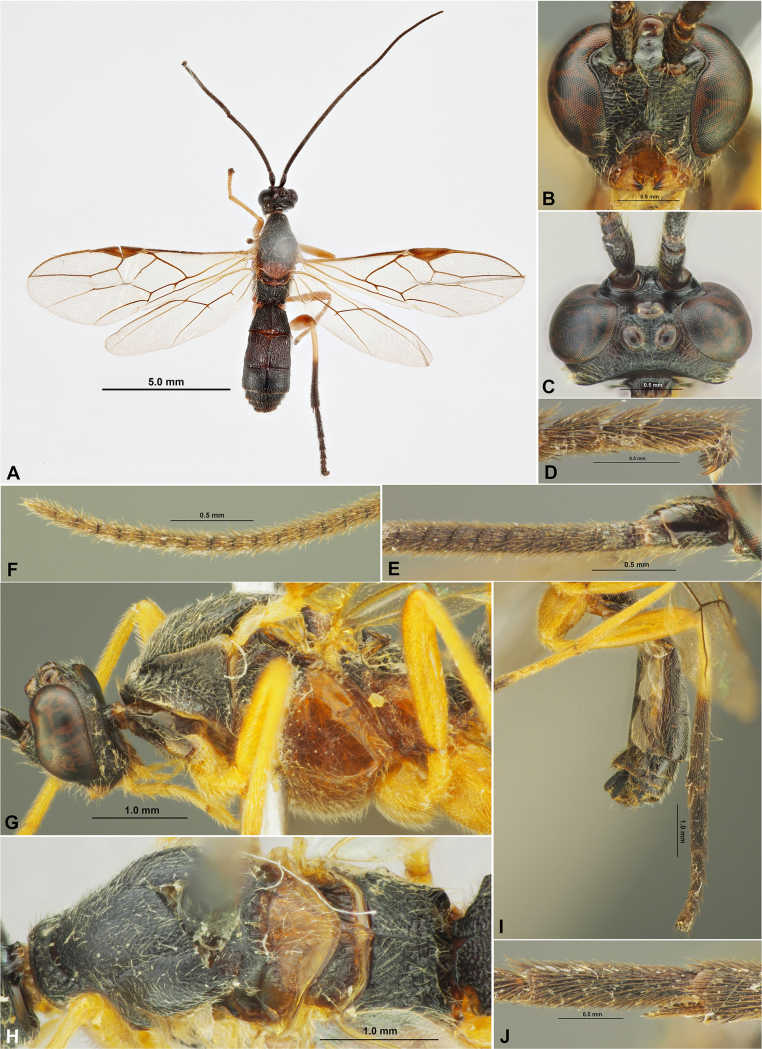
Aleiodes (Aleiodes) imberbis sp. nov., female, holotype. **A**. Habitus, dorsal view; **B**. Head, front view; **C**. Head, dorsal view; **D**. Claw of hind leg; **E**. Basal segments of antenna; **F**. Apical segments of antenna; **G**. Head and mesosoma, lateral view; **H**. Mesosoma, dorsal view; **I**. Hind leg; **J**. Apex of tibia, spurs and basitarsus of hind leg, inner side.

**Figure 12.**
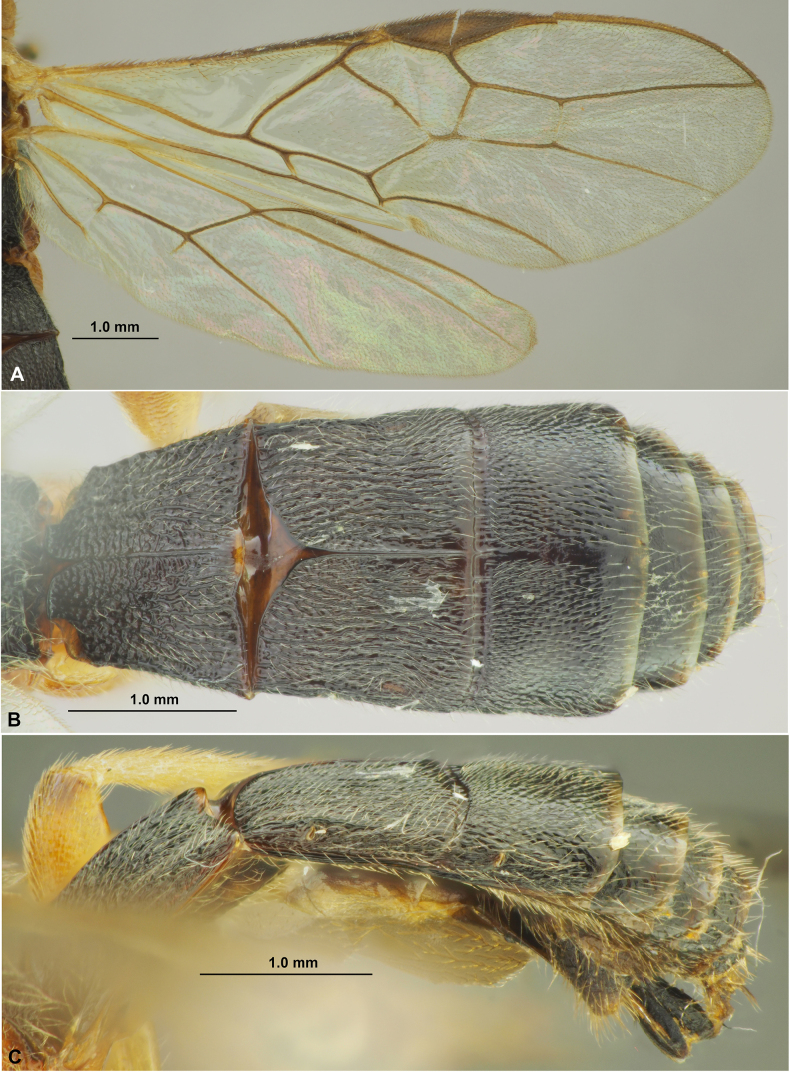
Aleiodes (Aleiodes) imberbis sp. nov., female, holotype. **A**. Wings; **B**. Metasoma, dorsal view; **C**. Metasoma, lateral view.

##### Description.

**Female. *Body*** length 8.7 mm; fore wing length 8.3 mm.

***Head*** width (dorsal view) 1.9× its median length, 1.2× width of mesoscutum. Occipital carina dorsally complete, but rather fine medially. Head behind eye (dorsal view) distinctly curvedly narrowed; transverse diameter of eye 2.7× length of temple. Ocelli strongly enlarged, arranged in almost equilateral triangle; POL 0.4× Od, almost 2.0× OOL; Od 8.0× OOL. Eye high, distinctly bean-shaped, 1.8× higher than broad (lateral view). Face convex, its width equal to height of face and clypeus combined, 0.7× height of eye. Hypoclypeal depression enlarged, circular, its width 1.4× distance from edge of depression to eye, ~ 0.4× minimum width of face. Malar space 0.2× height of eye, 0.8× basal width of mandible. Head below eyes (front view) distinctly and weakly-curvedly narrowed. ***Antennae*** thickened basally and slender apically, setiform, 68-segmented, approx. as long as body. Scape relatively long, widened distally, 1.7× longer than its maximum width. First flagellar segment 1.6× longer than its apical width, ~ 1.3× longer than second segment. Medial flagellar segments weakly elongated. Penultimate segment 1.6× longer than wide, 0.6× as long a apical segment. Apical segment acuminate and with distinct ‘spine’.

***Mesosoma*** 1.6× longer than high. Neck of prothorax short, convex, without pronope or longitudinal crest. Mesoscutum almost as wide as its medial length (dorsal view), distinctly and curvedly elevated above pronotum (lateral view). Prescutellar depression (scutal sulcus) relatively long, weakly curved posteriorly, ~ 0.4× as long as scutellum, with high median carina, coarsely rugose entirely. Precoxal sulcus present, but very shallow and wide, smooth. Metanotum without dorsal tooth (lateral view). Propodeum distinctly evenly curvedly oblique (lateral view), with wide and relatively flat posterolateral tubercles.

***Wings***. Fore wing 2.8× longer than its maximum width. Radial vein (r) arising weakly before middle of pterostigma. Radial (marginal) cell not shortened; metacarp (1-R1) 1.4× longer than pterostigma. First radial abscissa (r) very weakly curved, 0.9× as long as maximum width of pterostigma. Second radial abscissa (3-SR) 2.3× longer than first abscissa (r) and formed obtuse angle with it, ~ 0.45× as long as third radial abscissa (SR1), 1.65× longer than first radiomedial vein (2-SR). Second radiomedial (submarginal) cell medium-length, relatively wide, 1.9× longer than its maximum width, approx. as long as the narrow brachial (subdiscal) cell. Recurrent vein (m-cu) straight, 3.2× longer than second abscissa of medial vein (2-SR+M), 1.3× longer than first radiomedial vein (2-SR). Nervulus (cu-a) straight, inclivous, distinctly postfurcal, distance (1-CU1) between basal vein (1-M) and nervulus (cu-a) almost 2.0× longer than nervulus (cu-a); first abscissa of cubital vein (1-CU1) 0.5× its second abscissa (2-CU1). Most part of medial (basal) (except its narrow anterior stripe) and submedial (subbasal) cells, brachial (subdiscal) cell widely (except setose narrow medial elongate area) and narrow posterior area in discoidal (discal) cell glabrous and lustrous. In hind wing, radial (marginal) cell subparallel-sided at most basal part, but very weakly widened distally. Radial vein (SR) arising from costal vein (2-SC+R) closely to basal vein (r-m). Nervulus (cu-a) almost straight. First abscissa of mediocubital vein (M+CU) 1.2× longer than second abscissa (1-M). Recurrent vein (m-cu) present, straight, very short, pigmented, antefurcal. Medial (basal) cell of hind wing very sparsely setose, mostly glabrous.

***Legs***. Fore femur 5.6× longer than maximum width. Hind trochantellus 1.1–1.2× longer than hind trochanter (measured on their lower margins). Hind femur 4.0× longer than its maximum width. Hind tibia weakly widened posteriorly, without comb of dense setae on its inner posterior margin. Longest inner spur of hind tibia ~ 0.4× as long as hind basitarsus. Hind tarsus thickened, 0.9× as long as hind tibia. Hind basitarsus 0.8× as long as second to fifth segments combined. Second segment of hind tarsus 0.4× as long as basitarsus, approx. as long as fifth segment (without pretarsus). Claw densely pectinate by long dark pectens.

***Metasoma***. Tergites behind third one not strongly protruding. First tergite strongly and almost linearly widened basally, then distinctly and linearly widened towards its posterior margin; length of tergite 0.8× its maximum posterior width; its posterior width 1.7× width at level of dorsope, 2.5× its anterior (basal) width. Basal area of first tergite rather narrow and short, semi-round, with single pointed break medio-posteriorly. Medial length of second tergite 0.8× its anterior width; 1.2× length of third tergite; its basal area wide and rather long; first and second tergites with complete and distinct carina. Suture between second and third tergites distinct, relatively wide, almost straight and crenulate. Ovipositor sheath 0.4× as long as hind basitarsus, ~ 0.3× as long as first tergite.

***Sculpture and pubescence***. Vertex reticulate-rugose; temple densely transverse striate with reticulation; frons mostly smooth; face transversely striate, rugulose-punctate medially. Mesoscutum and scutellum entirely densely granulate with sparse punctation at least partly. Mesopleuron mostly smooth, upper anterior area densely coarsely reticulate-rugose. Propodeum entirely coarsely rugulose-reticulate with distinct and almost complete medial carina. Hind coxa almost smooth dorsally, densely and finely punctate laterally; hind femur rather densely and finely punctate. First and second metasomal tergites entirely densely and coarsely striate with dense rugulosity between striae, third tergite mostly densely reticulate-punctate, but almost smooth posteriorly; anterior area of first and second tergites smooth; only first and second tergites with complete medial carinae. Following tergites smooth.

***Colour***. Body mostly black; scutellum, axillae, most part of mesopleuron and all metapleuron pale reddish brown. Antennae entirely black. Palpi yellow. Legs mostly yellow to brownish yellow, basal half of hind tibia pale yellow, apical half of hind tibia, hind tarsus entirely and fifth segments of fore and middle tarsi black. Wing subhyaline; pterostigma widely brown, paler medially.

**Male**. Unknown.

##### Comparative diagnosis.

This new species is similar to Aleiodes (A.) krasheninnikovi Belokobylskij, 1996 from the Russian Far East (Belokobylskij 1996), but distinctly differs from it by having the ocelli large, POL 0.4× Od (smaller, POL 0.6× Od in *A. krasheninnikovi*), basal half of the fore wing mostly glabrous and shiny (densely setose and mat in *A. krasheninnikovi*), anterior triangle area of the second tergite large (absent in *A. krasheninnikovi*), claw densely and widely pectinate (not pectinate in *A. krasheninnikovi*), basal half of hind tibia pale yellow and its apical half black (entirely pale reddish brown in *A. krasheninnikovi*), and body mostly black (entirely pale reddish brown in *A. krasheninnikovi*).

Also *Aleiodes imberbis* sp. nov. is superficially similar to A. esenbeckii ssp. dendrolimi (Matsumura, 1926) from the East Palaearctic and East Oriental regions (Kirichenko et al. 2024), but differs distinctly from the latter by having the basal half of the fore wing mostly glabrous and shiny (densely setose and mat in A. esenbeckii ssp. dendrolimi), mesopleuron mostly smooth (entirely densely punctate-granulate in A. esenbeckii ssp. dendrolimi), nervulus (cu-a) of the fore wing inclivous (declivous in A. esenbeckii ssp. dendrolimi), first abscissa of the cubital vein (1-CU1) of fore wing 0.5× its second abscissa (2-CU1) (approx. equal to or slightly shorter in A. esenbeckii ssp. dendrolimi), anterior triangle area of the second tergite large (absent in A. esenbeckii ssp. dendrolimi), claw densely and widely pectinate (not pectinate in A. esenbeckii ssp. dendrolimi), and basal half of hind tibia pale yellow and its apical half black (entirely dark reddish brown in A. esenbeckii ssp. dendrolimi).

##### Etymology.

This species is named after *imberbis* (Latin for beardless), referring to the most basal part of the fore wing, which is glabrous and shiny.

##### Host.

Unknown.

##### Distribution.

Korean Peninsula.

## Supplementary Material

XML Treatment for Aleiodes

XML Treatment for Aleiodes (Arcaleiodes) monochromus

XML Treatment for Aleiodes (Chelonorhogas) pseudalbitibia

XML Treatment for Aleiodes (Chelonorhogas) rufoniger

XML Treatment for Aleiodes (Aleiodes) crassicornis

XML Treatment for Aleiodes (Aleiodes) heterogamoides

XML Treatment for Aleiodes (Aleiodes) imberbis

## Additional information

### Artificial Intelligence (AI) use

The authors accept full responsibility for the content of the manuscript, including the disclosure of any use of AI.

No AI tools were used in the preparation of this manuscript.

### Author ORCIDs

S.A. Belokobylskij https://orcid.org/0000-0002-3646-3459

D. Ku https://orcid.org/0000-0002-6274-6479

H.-R. Lee https://orcid.org/0000-0002-3860-8850

### Data availability

All of the data that support the findings of this study are available in the main text.
